# Selenium (IV) and Sulphur (VI) as Elements Modifying Plant Quality: Content of Selenium and Sulphur Forms in Wheat

**DOI:** 10.3390/molecules31010160

**Published:** 2026-01-01

**Authors:** Marzena S. Brodowska, Magdalena Kurzyna-Szklarek, Mirosław Wyszkowski

**Affiliations:** 1 Department of Agricultural and Environmental Chemistry, University of Life Sciences in Lublin, Akademicka 15 Str., 20-950 Lublin, Poland; 2 Department of Agricultural and Environmental Chemistry, University of Warmia and Mazury in Olsztyn, Łódzki 4 Sq., 10-727 Olsztyn, Poland

**Keywords:** selenium, sulphur, spelt wheat, common wheat

## Abstract

In order to achieve high-quality yields, it is essential to provide plants with the necessary nutrients, including selenium (Se) and sulphur (S), to meet their nutritional requirements. This study aimed to determine the effect of selenium (0, 10 and 20 g Se ha^−1^) and the date of its application (in the tillering phase and in the stem elongation phase) and sulphur application (0, 15 and 30 kg S ha^−1^) on the content of selenium and various forms of sulphur (total sulphur, sulphate sulphur and organic sulphur) and the N:S ratio in winter spelt wheat and winter common wheat. The research hypothesis assumed that different doses of selenium and sulphur and the timing of their application would have a beneficial effect on the Se and S content in the grain and straw of spelt wheat and common wheat. Selenium fertilisation significantly increased the content of this element in the grain of spelt wheat and common wheat. The concentration of selenium was also influenced by the timing of its application in the plant growth environment. However, the dose of selenium and the timing of its application were not associated with significant changes in the content of both forms of sulphur in the tested plants. The experimental factors used did not contribute to the achievement of selenium levels toxic to humans and animals. The presence of sulphur in the growth environment of spelt wheat and common wheat was associated with an increase in the content of both total sulphur, sulphate sulphur and organic sulphur in their grain and straw, especially in spelt wheat straw by an average of 17%, 29% and 23%, respectively, and in common wheat straw by 26%, 18% and 57%, respectively. The sulphur content in the plant growth environment was not associated with a change in the selenium content in the grain of the tested plants. The results of our study suggest that the optimal dose of selenium for biofortification of humans and animals is 20 mg Se ha^−1^ on clay soil, applied during the stem elongation phase of spelt and common wheat. Biofortification of wheat with selenium and sulphur is a good method of supplementing deficiencies of this element in the human diet.

## 1. Introduction

Since the 1960s, selenium (Se) fertilisation of crops has attracted the interest of researchers around the world [1,2]. Most of the experiments conducted involved the use of various selenium salts in the form of soil fertilisers or in combination with phosphorus and nitrogen fertilisers [3,4]. The method of application, but also the fertilisation strategy, are considered key issues in selenium fertilisation, as the responses of crops depend on the fertiliser dose, Se speciation, and the method and timing of application [5,6]. Knowledge of the impact of fertiliser type, strategy and timing allows the development of sustainable fertilisation strategies targeted at site-specific causes of Se deficiency [7]. It is believed that crops respond more strongly to fertilisers containing selenate (VI) compared to fertilisers with selenite (IV), mainly due to its greater solubility and availability in plants [5]. The Se level in plants increases quite rapidly after fertilisation, but also decreases when the Se level in the soil solution is reduced. Its half-life is estimated to be 21 to 80 days in grassland ecosystems [8,9], but other researchers have observed a positive response in crops even 3 years after application [2]. Foliar application is considered to be almost twice as effective as granular soil fertilisers or seed enrichment treatments [6,10].

The average Se level in wheat in the United Kingdom and Scandinavian countries ranges from 7 to 22 μg kg^−1^ d.m. [11], and the nutritional value considered adequate for animal and human health is approximately 50 to 100 μg kg^−1^ [12]. Therefore, an increase in Se levels in crops of approximately 50% as a result of optimal fertilisation is considered sufficient to prevent Se deficiency in humans and animals. Assuming a 10% recovery of selenium from fertilisers [13], a wheat yield of approximately 7 tonnes ha^−1^ and the fact that only about 50% of the added selenium ends up in the grains, the recommended selenium dose is approximately 4 to 13 g Se ha^−1^. This is considered sufficient to achieve the required nutritional value. This estimated fertiliser dose is also within the typical fertiliser doses recommended by Finnish and Canadian guidelines [14,15,16,17]. Other authors have reported slightly higher recovery rates, ranging from 14 to 18% in cereals [18], 8 to 32% in wheat [5] and 5 to 25% in cereal crops [19]. In contrast, the results of Tolu et al. [9] and Guo et al. [3] suggest that even more than 90% of the Se applied is unavailable and cannot be taken up by crops. This low level of Se recovery when applied foliar can be partly explained by the fact that it is usually applied during the growing season before intensive leaf development, resulting in a combination of Se applied to the leaves and to the soil. Nevertheless, foliar application of Se is considered to be on average 8 times more efficient than soil application, which is why foliar application is preferred over soil application [6,19].

Fertilisation with macronutrients can affect the Se content in crops [2]. This is related to the competition between SO_4_^2−^ and PO_4_^3−^ ions and Se in terms of uptake by plants [20,21]. In the case of selenium fertilisers, a strong tendency to reduce the response of crops to Se fertilisation with increasing doses of S and P has been observed. The use of selenite (IV) fertilisers weakens this tendency. The observed reduction in Se uptake due to sulphates is explained by ion competition for transport pathways in plant roots [22]. The reducing effect of sulphate ions has been reported in previous studies [23,24,25,26].

Selenium and sulphur (S) are chemically very similar, which affects their uptake and metabolism in plants. Both elements occur in the soil mainly in the form of anions—selenates and sulphates—and compete for the same transport systems in the roots [27,28]. Selenium uptake occurs mainly through sulphate transporters in the plasma membrane of roots, and its further conversion follows the sulphur metabolic pathway [29]. High sulphur availability in the rhizosphere limits selenate uptake due to competitive antagonism at the transporter level [30,31]. On the other hand, sulphur deficiency may increase selenium absorption, as plants under sulphur deficiency conditions intensify the expression of sulphate transporters, which promotes the influx of selenates [30]. This phenomenon is observed, among others, in *Arabidopsis thaliana*, where an increase in sulphate concentration in the substrate reduces Se accumulation in shoots, while improving biomass growth [30]. After entering the plant, selenium enters the sulphur assimilat—ion pathway, where enzymes such as ATP-sulfurylase and APS reductase act on both sulphates and selenates [32]. This biochemical convergence allows selenium to replace sulphur in amino acids, forming selenocysteine and selenomethionine, which are incorporated into proteins [28]. However, such substitution often disrupts protein functions, which at high Se concentrations leads to toxicity [27].

Recent studies confirm that sulphur fertilisation strategies significantly affect the effects of selenium biofortification. For example, adequate sulphur supply reduces Se accumulation in wheat grain, while sulphur deficiency increases selenium uptake but may limit plant growth [33,34]. This indicates the need to balance S and Se supply in order to achieve optimal crop quality and health benefits.

Selenium and sulphur play an irreplaceable role in wheat metabolism, improving physiological processes, protein synthesis and stress resistance. Adequate selenium fertilisation enhances the activity of antioxidant enzymes, accelerates root development and increases the accumulation of selenium in organic forms—mainly selenomethionine—in the grain [35]. Sulphur, on the other hand, is crucial for the synthesis of sulphur amino acids and proteins, enhancing photosynthetic efficiency and supporting yield growth [36,37]. Selenium deficiency can lead to reduced resistance to environmental stress, weakened antioxidant protection and an increased risk of nutritional deficiencies in consumers. On the other hand, excess selenium can be toxic, but intelligent dosing—according to current research—allows this risk to be avoided [38]. Sulfur deficiency results in reduced protein content and nitrogen metabolism disorders, while adequate supply promotes synergistic interactions with nitrogen and selenium, while supporting the efficient use of nutrients [37,39]. As a result, the optimal use of selenium and sulphur as part of integrated fertilisation contributes to improved plant growth, grain quality and nutritional value.

Research indicates clear differences between spelt wheat (*Triticum spelta* L.) and common wheat (*Triticum aestivum* L.) in terms of metabolism and micronutrient uptake, which affects the effectiveness of biofortification strategies. Field experiments have shown that spelt wheat achieves higher selenium concentrations in the grain, which correlates with morphological characteristics such as greater plant height, indicating different accumulation capacities compared to common wheat [40]. In addition, sulphur-selenium application increased the copper and zinc content mainly in spelt straw, confirming its greater susceptibility to the synergistic effect of micronutrients [41]. In turn, common wheat reacts more strongly to the availability of protein nitrogen, especially when interacting with selenium and sulphur, as confirmed by data showing a significant increase in mineral nitrogen in straw after fertilisation [37]. Additionally, genetic studies highlight differences between species—spelt shows better selenium accumulation potential in traditional varieties, which is a promising target for selection and breeding focused on nutritional value [40]. The conclusion is that metabolic and adaptive differences between spelt and common wheat should be taken into account when designing fertilisation and biofortification programmes, which will enable optimised growth support, stimulation of the accumulation of valuable components and increased nutritional value of crops.

Recent studies indicate the growing importance of selenium and sulphur in agriculture, both in the context of biofortification and improving plant resistance to environmental stress. The use of selenium in appropriate doses and at appropriate times increases its accumulation in cereal grains while minimising the risk of toxicity [36]. Increasing attention is being paid to innovative forms of application, such as selenium nanoparticles, which have multidirectional effects, including improved antioxidant properties and reduced heavy metal bioaccumulation [42,43]. At the same time, research on molecular mechanisms confirms the role of selenium in regulating secondary metabolism and alleviating metal stress [38,39]. In the case of sulphur, its presence in the plant growth environment supports photosynthesis, sulphur amino acid synthesis and improves crop quality, which, in combination with selenium, creates synergistic biofortification strategies [44]. In the face of global selenium and sulphur deficiencies in soils, the integration of fertilisation with these elements appears to be a key element of sustainable agriculture, supporting food security and human health.

The research hypothesis was that varying doses of selenium and sulphur and the timing of the application of the former would have a beneficial effect on the Se and S various forms (total-S, sulphate S and organic S) content in the grain and straw of spelt wheat and common wheat. With this in mind, studies were conducted to determine the effect of selenium and the date of its application and sulphur on the content of selenium and various forms of sulphur (total sulphur, sulphate sulphur and organic sulphur) and the N:S ratio in winter spelt wheat and winter common wheat. The analysis of different sulphur fractions—total, sulphate and organic—is crucial for understanding the role of this element in plant metabolism. Sulphates are a form available to plants and are the main substrate for the synthesis of sulphur amino acids, such as cysteine and methionine, which affect protein metabolism and immune mechanisms [45]. The permanent organic form of sulphur, bound to organic matter, acts as a buffer and constitutes a susceptibility reserve, gradually mineralised by microorganisms, which helps maintain the balance of sulphates in the soil [46]. Studies also confirm that the sulphur content in the soil affects microbial diversity and activity, which determines the dynamics of transformations and the availability of sulphur to plants [47]. As a result, a comprehensive analysis of sulphur fractions enables precise fertiliser dosing and optimisation of growth conditions, which translates into an effective and sustainable fertilisation strategy.

## 2. Results and Discussion

### 2.1. Selenium of Wheat

The selenium content in spelt and common wheat grains (Table 1a,b) was consistent with the data in the literature [34,48].

However, in most cases, the control plants (without selenium fertilisation) had a lower content than 0.1 mg kg^−1^, which is considered optimal for nutritional purposes [40]. The selenium content in cereal grains was also studied by Wang et al. [49], who showed that the average content of this element was below 0.1 mg kg^−1^, while the average selenium content in spring wheat grain was 0.066 mg kg^−1^. In Slovakia, the average selenium content in wheat was even lower, at 0.029 mg kg^−1^ d.m. [50].

Selenium fertilisation resulted in an average 4-fold (lower dose) and more than 5-fold (higher dose) increase in its content in common wheat grain, as well as in a 3-fold and in a 4-fold increase in Se content in spelt wheat grain, compared to objects without selenium in the fertiliser dose (Table 1b). Selenium fertilisation at a later date resulted in a twofold increase in Se content in common wheat and spelt wheat grain. Sulphur fertilisation reduced the selenium content of common wheat grain by an average of 17% and 25%, respectively.

The selenium content in spelt and common wheat straw (Table 2a,b) was very low. No significant changes in its content were observed under the influence of the experimental factors used.

In studies by Manojlović et al. [51], selenium fertilisation was associated with a two- to five-fold increase in its content in winter wheat grain. Foliar application of selenium also resulted in a significant increase in its content in maize and bean seeds [52]. Ramkissoon et al. [18] and Wang et al. [53] report an increase in selenium concentration in wheat grain to 0.350 mg kg^−1^ after applying a dose of 30 g ha^−1^. Radawiec et al. [48] and Ducsay et al. [50,54] reported an increase in selenium content to 0.445 mg kg^−1^ after applying a dose of 10 g ha^−1^ of selenium. Wang et al. [55] found that foliar fertilisation with sodium selenite (IV) at a dose of 6 and 12 g Se ha^−1^ is associated with an increase in Se content in winter wheat grain from 0.042 to 0.067 mg kg^−1^ and from 0.065 to 0.180 mg kg^−1^ d.m., respectively. Research by Klikocka et al. [56] and Yeasmin et al. [21] reports that fertilisation with sulphur at a dose of 50 kg ha^−1^ results in an increase in selenium content in spring wheat grain.

It is believed that selenium doses ranging from 10 to 20 g ha^−1^ Se allow biofortification goals to be achieved [50,51,52,57]. Studies evaluating the effect of Se application at doses ranging from 0 to 25 g ha^−1^ showed an increase in Se content in plants [58,59]. In our own studies, doses consistent with those mentioned above were used, but they did not result in an excessive increase in selenium content in wheat grains compared to the above studies. The target desired range of selenium content in cereal grains after biofortification, at which there is no risk of toxic effects, is 0.1 to 1.0 mg kg^−1^ [60,61,62]. The studies conducted allowed us to obtain selenium content in grain within the above range.

Furthermore, when comparing the results of our own research to studies reporting high selenium supply without toxic effects and at the same time ensuring the desired selenium content in the blood [63], they are similar. The highest selenium content in spelt wheat grain, 0.38 mg kg^−1^ Se, was found for the total combination of the highest sulphur dose and the highest selenium dose in spelt wheat grain applied on the second date, while in common wheat, the highest content was 0.30 mg kg^−1^ and was found in the combination of the highest dose of selenium applied in the second period without sulphur fertilisation. The results obtained demonstrate the effective implementation of agrotechnical biofortification aimed at increasing the selenium content in the grain of test plants, but without toxic effects. In straw, however, no positive effect of selenium fertilisation on its content was observed, therefore straw should not be considered a source of selenium for the biofortification of animals. The research conducted may help fertiliser manufacturers select the best fertilisers and Se levels to ensure the safe inclusion of Se in the food chain in the future, thereby improving Se intake by humans and animals [64].

According to Ducsay et al. [50], the best time to introduce selenium into wheat is during the tillering (BBCH 20–29) and stem elongation (BBCH 30–39) stages. Zhang et al. [36]. According to their research, the best method of fertilising wheat to enrich it with selenium is foliar or soil fertilisation around the BBCH 31 growth stage. Our own research is consistent with these theories, as in both test plants, higher selenium content was found in plants fertilised with Se during the stem elongation stage (BBCH 31–34).

### 2.2. Total Sulphur of Wheat

Wheat is classified as a plant with relatively low sulphur requirements, but a deficiency of this element can result in poorer nitrogen management and reduced grain quality [65,66]. The literature contains reports of no difference in sulphur content between grain and straw [67], higher total sulphur content in straw than in grain [68], and those in which the sulphur content in straw is lower than in grain [69]. The results of our own research on the sulphur content in both spelt and common wheat grain and straw [Table 3a,b] are consistent with the research of other authors [34].

The application of a lower dose of selenium resulted in an average 7% increase in total sulphur content, while a higher dose resulted in a 10% decrease in spelt wheat grain [Table 3b]. The application of both lower and higher doses of selenium to the common wheat growth environment was associated with an increase in total sulphur content in the grain by an average of 16–17%. Later selenium fertilisation resulted in an 8% decrease in total sulphur content in spelt wheat grain. The total sulphur content in spelt wheat grain increased by an average of 5% as a result of applying a lower dose of sulphur to the plant growth environment. An increase in the total sulphur content in common wheat grain, compared to control objects without sulphur in fertilisers, was observed in plants fertilised with a lower dose of sulphur.

The effect of applying selenium and sulphur on the total sulphur content of wheat straw is illustrated in Table 4a,b. Selenium fertilisation in most cases resulted in a decrease in total sulphur content in spelt wheat straw by an average of 5% and 15%, respectively [Table 4b]. The application of selenium at a lower dose was associated with a 24% decrease in total sulphur content in common wheat straw, and at a higher dose with a 20% increase. Fertilisation with a higher dose of sulphur resulted in an increase in total sulphur content in spelt wheat straw by an average of 17% and in common wheat straw by 26%.

Studies by Hrivna et al. [68] and Liu et al. [66] confirm an increase in total sulphur content in winter wheat grain as a result of sulphur fertilisation. In studies by Rodrigo et al. [70], the total sulphur content in barley did not change under the influence of selenium fertilisation, even at a dose of 500 g Se ha^−1^. Similarly, in the studies by Yeasmin et al. [21], the content of the elements studied, including sulphur, was not sensitive to selenium fertilisation. However, in the studies by Silva et al. [71] and Saeed et al. [33], the sulphur content decreased in the test plants under the influence of selenium fertilisation.

### 2.3. Sulphate Sulphur of Wheat

The sulphate sulphur content in both the grain (Table 5a,b) and straw (Table 6a,b) of spelt wheat and common wheat was at the level most commonly found in the literature [58,72].

Fertilisation with selenium at a lower dose was associated with an average 6% increase in sulphate sulphur content only in common wheat grain (Table 5b). Fertilisation with a lower sulphur dose resulted in an average 26% increase in sulphate sulphur content, and a higher dose resulted in a 56% increase in spelt wheat grain. Sulphur fertilisation was associated with a significant reduction in the sulphate form of sulphur in common wheat grain by 19% and 13%, respectively.

The application of selenium to the growth environment of common wheat was associated with a decrease in sulphate sulphur content by an average of 21% and 11% in straw, respectively (Table 6b). Sulphur fertilisation resulted in an increase in sulphate sulphur content in spelt and common wheat straw in most cases. The application of a higher dose of S was associated with an average increase of 29% in spelt wheat straw and 18% in common wheat straw.

According to Schulte and Kelling [73], the sulphur content in plants is significantly influenced by the abundance of this element in the soil. Research by Pokhrel et al. [74] confirms an increase in sulphate sulphur content in rapeseed under the influence of sulphur fertilisation. Gil-Díaz et al. [75] noted this relationship in spring barley biomass. Studies by Qiu et al. [76] and Lima Gomes et al. [77] report an increase in sulphate sulphur content in the plants studied—in barley and tomatoes, respectively—under the influence of selenium fertilisation.

### 2.4. Organic Sulphur of Wheat

Organic Sulphur content of Wheat was presented in Table 7a,b (grain) and 8a,b (straw). Selenium fertilisation was associated with a significant increase in organic sulphur content of 11% was observed in spelt wheat grain under the influence of fertilisation with a lower dose (Table 7b). Selenium fertilisation caused an increase in the organic sulphur content in common wheat grain, with the difference that this reaction was observed after the application of both doses (an average increase of 23% and 33%, respectively). The second application of selenium was associated with a 10% decrease in the organic sulphur content of spelt wheat grain. An average 14% decrease in organic sulphur content in spelt wheat grain was observed after applying a higher dose of sulphur, compared to plots without sulphur in fertilisers. Fertilisation with a lower dose of sulphur resulted in a significant and clear increase in organic sulphur content (by 25%) in common wheat grain.

A significant increase in organic sulphur content in spelt wheat grain under the influence of a lower selenium dose may be related to the induction of sulphur metabolic pathways by selenium, due to their chemical similarity [5,78]. In the case of common wheat, an increase in organic sulphur content was observed after the application of both selenium doses, indicating a stronger interaction between these elements in this species [21]. The reduction in organic sulphur content in spelt wheat grain associated with the delay in selenium application may be due to a shift in the phase of activity of the enzymes responsible for the synthesis of sulphur amino acids [64].

The lack of increase in organic sulphur content in spelt wheat grain under the influence of higher sulphur fertilisation is consistent with observations that excessive sulphur doses may not bring the expected effects in terms of protein quality improvement and may even lead to a decrease in the content of sulphur compounds in the grain [68,79]. This may be due to disturbances in the metabolism of sulphur amino acids when there is an excess of this element [66]. On the other hand, the effect of a lower sulphur dose, manifested by a significant increase in the organic sulphur content in common wheat grain, confirms the importance of optimising fertiliser doses in order to avoid antagonism [65].

A higher dose of sulphur resulted in an average 14% increase, and a higher dose of selenium resulted in a 23% decrease in the organic sulphur content of spelt wheat straw (Table 8b). Sulphur fertilisation caused an average 29% and 57% increase in the organic sulphur content of common wheat straw, respectively. Fertilisation with a higher dose of selenium and a delay in its application resulted in an increase in the organic sulphur content of common wheat straw.

In spelt wheat straw, a higher dose of sulphur resulted in an increase in organic sulphur content, while a higher dose of selenium resulted in a decrease. This confirms the hypothesis of competition between these elements in transport pathways [21,34]. In common wheat, sulphur fertilisation caused a marked increase in the organic sulphur content of straw, and delaying the application of selenium further increased this effect, which may be related to a longer period of accumulation of sulphur compounds in vegetative tissues [34].

### 2.5. N:S Ratio of Wheat

The N:S ratio (total nitrogen content to total sulphur content) in the generative organs of crops is an indicator that is very often used to diagnose sulphur deficiencies in plants [80,81]. The accumulation of non-protein forms of nitrogen in plants (amides, amino acids and nitrates) is promoted by an increase in the N:S ratio. On the other hand, a decrease in this ratio leads to the accumulation of inorganic sulphur compounds [82]. Various theories regarding the optimal value of this ratio can be found in the literature. Sedlár et al. [83] report that in climatic conditions, wheat with the best technological value is obtained with an N:S ratio ranging from 9.5–10:1 to 17:1. The highest cereal yields are obtained when the ratio is 15:1 [84], while the critical value of the ratio is 17:1 [85]. In our own research, the values of the discussed indicator were in most cases within the given ranges (Table 9a,b and Table 10a,b).

Fertilisation with a lower dose of selenium resulted in an average 9% reduction in the C:N ratio, while a higher dose resulted in a 6% increase in spelt wheat grain (Table 9b). Selenium fertilisation was associated with a narrowing of the N:S ratio by 13% and 16%, respectively, in common wheat grain. A higher average N:S ratio in spelt wheat grain was obtained in plots fertilised with selenium on the second application date. In spelt wheat grain, fertilisation with a higher dose of sulphur led to an average 5% reduction in the C:N ratio in most cases.

The application of selenium resulted in an increase in the N:S ratio in spelt wheat straw by an average of 6 and 10%, respectively (Table 10b). Fertilisation with selenium and timing of its application did not clearly differentiate the N:S ratio values in common wheat straw. Fertilisation with a higher dose of sulphur resulted in a 10% reduction in the N:S ratio in spelt wheat straw. The presence of both lower and higher doses of sulphur in the plant growth environment was associated with a reduction in the N:S ratio by an average of 11% and 31%, respectively, in common wheat straw.

The results of our own research are consistent with the studies by Roa et al. [65] and Klikocka et al. [86], who report a narrowing of the N:S ratio as a result of the use of sulphur in fertilisation, which results in a more favourable nutritional value of plants. Arrigoni et al. [87], on the other hand, obtained the highest value of this parameter in wheat grain fertilised with nitrogen and sulphur. Despite the fact that wheat is not a plant with a high sulphur requirement, its deficiency can lead to reduced nitrogen utilisation and a narrowing of the N:S ratio [80]. The experiment by Ullah et al. [88], which examined different types of sulphur fertilisers, proved that elemental sulphur had the least favourable effect on the N:S ratio, while ammonium sulphate had the most positive effect. Chien et al. [89], Pimentel et al. [90] and LaBarge [72] also found a positive effect of ammonium sulphate on the total sulphur content in the tested plants, as well as on the N:S ratio.

The yield and quality of wheat useful parts, including the content of selenium and sulphur compounds, depend on optimal doses of mineral fertilisers [91,92], which should also be used in quantities that do not affect the environment [93]. The results of our own 3-year field research presented above, as well as the scientific discussion, show that the application of selenium and sulphur has a beneficial effect on the chemical composition of spelt wheat and common wheat. It seems necessary to conduct research with other plant species, adjusting doses to their nutritional and fertiliser requirements.

In summary, it can be concluded that the interactions between selenium and sulphur result from their chemical and biochemical similarities—both elements belong to the same group of the periodic table and use similar transport and assimilation pathways in plants. Selenium in the form of selenite (SeO_3_^2−^) is taken up by plants mainly through sulphate transporters, leading to enzymatic competition at the reduction stage in chloroplasts [38]. After entering the cell, selenium undergoes a reduction pathway analogous to sulphur, participating in the biosynthesis of sulphur amino acids such as selenocysteine and selenomethionine [45]. This competition explains why an increased supply of sulphur can limit the accumulation of selenium in grain, while not significantly affecting its content in straw—intensive protein synthesis occurs in grain, requiring sulphur amino acids, which promotes the incorporation of selenium into organic structures.

The different reactions of spelt and common wheat result from different mechanisms of nitrogen and sulphur metabolism regulation and differences in the expression of sulphate transporters and reductase enzymes [40]. Common wheat, characterised by higher protein synthesis intensity in the grain, has a greater ability to incorporate selenium into organic forms, which explains the higher increase in its concentration in the grain compared to spelt. In straw, on the other hand, structural rather than metabolic processes dominate, which is why Se–S interactions are less pronounced. The developmental phase is also of key importance—the application of selenium in the stem elongation phase (BBCH 31–34) coincides with the intense activity of sulphate-reducing enzymes, which promotes competition and the incorporation of selenium into organic metabolites [39].

Discrepancies in the observed effects of selenium and sulphur fertilisation may result from several biological and environmental factors. Firstly, the lack of clear changes in the sulphur fraction content when selenium is applied may be related to differences in uptake mechanisms—sulphates are actively taken up by specialised transporters, while selenite only partially competes with them, which limits the impact of selenium on the sulphur pool in the plant [45]. Secondly, the observed differences between grain and straw may result from the preferential targeting of sulphur amino acids and their selenium analogues to generative organs, where intensive protein synthesis determines greater selenium incorporation [38]. Structural processes dominate in straw, which are less dependent on sulphur and selenium metabolism, which explains the lack of clear interactions. The differences between spelt and common wheat may, in turn, be the result of different expression of sulphate transporters and reductase enzymes, as well as differences in the rate of translocation of components to the grain [40]. Alternatively, the effect of the timing of selenium application may be modulated by environmental conditions (humidity, temperature) that determine the activity of selenite and sulphate reducing enzymes, leading to variable biofortification efficiency [39].

## 3. Materials and Methods

### 3.1. Research Methodology

This research was conducted on the basis of a three-year field experiment carried out at the Experimental Station of the University of Life Sciences in Lublin, located in Czesławice (51°18′23′′ N, 22°16′02′′ E), Poland. The research was conducted between 2015 and 2018. The research was conducted on plots located in the same place on soil with the same properties. The experiment was set up on clay soil according to the IUSS Working Group WRB [94] classification, formed from loess deposits. The chemical composition of the soil was included in the first, already published article on this experiment [34]. The tested soil was characterised by a slightly acidic reaction (pH_KCl_ - 6.3) and a moderate mineral nitrogen content of 0.83 mg NH_4_-N kg^−1^ d.m. and 22.39 mg NO_3_-N kg^−1^ d.m. It was distinguished by a high concentration of phosphorus (105.5 mg P kg^−1^ d.m.) and magnesium (59.0 mg kg^−1^ d.m.), while the sulphur content in the form of sulphate was average and amounted to 10.4 mg SO_4_ kg^−1^ d.m. A similar average level was recorded for potassium, which amounted to 134.5 mg K kg^−1^ d.m. These properties indicate a soil rich in macroelements, with a simultaneous need to monitor the availability of sulphur in the context of plant biofortification. Two model plant species were used in the study: winter spelt wheat (*Triticum spelta* L.) Rokosz variety (Hodowla Roślin Strzelce Sp. z o.o., Strzelce, Poland) and winter common wheat (*Triticum aestivum* L.) Astoria variety (Poznańska Hodowla Roślin, Tulce, Poland).

**Figure 1:**
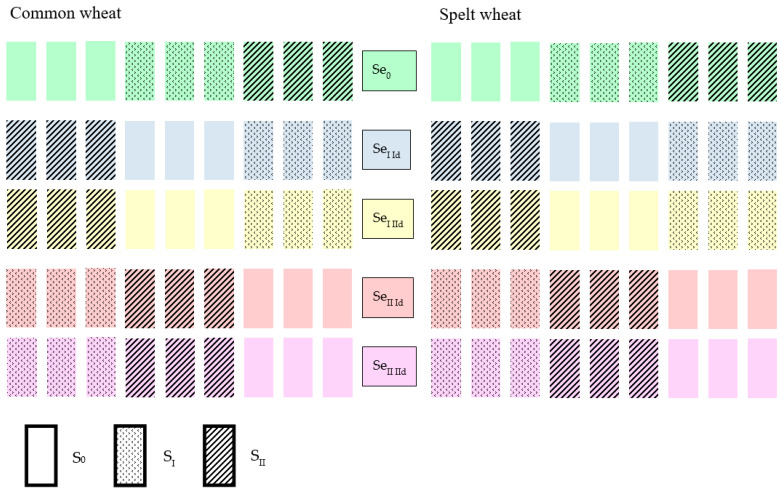
Scheme of the field experiment; Id—1st selenium application date, IId—2nd selenium application date.

The experiment was designed as a three-factor system in a factorial design. The first factor included three levels of sulphur application: S_0_—0 kg S ha^−1^, S_1_—15 kg S ha^−1^ and S_2_—30 kg S ha^−1^. The second factor consisted of three levels of selenium supplementation: Se_0_—0 g Se ha^−1^, Se_1_—10 g Se ha^−1^ and Se_2_—20 g Se ha^−1^. The third factor referred to two selenium application dates, i.e., in the tillering phase (BBCH 22–24) and in the stem elongation phase (BBCH 31–34). Factors S and Se were introduced at three levels, while the application date was introduced at two levels, which enabled the analysis of interactions between the studied variables. The field experiment design is shown in Figure 1.

The plants were sown in the first ten days of October and harvested in the second ten days of August, at the stage of full physiological maturity. Sulphur was applied before sowing in the form of ammonium sulphate ((NH_4_)_2_SO_4_), while selenium was applied in the form of sodium selenite (IV) (Na_2_SeO_3_). Sodium selenite (IV) [Na_2_SeO_3_] is preferred in biofortification due to its high solubility, stability in the soil environment and easy reduction to organic forms in plants, which promotes the synthesis of selenomethionine and selenocysteine [38]. Ammonium sulphate [(NH_4_)_2_SO_4_] provides sulphur in the form of sulphate, which is a direct substrate for the biosynthesis of sulphur amino acids, while also introducing ammonium nitrogen, supporting protein metabolism [47]. Both forms are consistent with the principles of green chemistry—they are characterised by high efficiency of ingredient use, low mobility in the environment and minimal risk of ecotoxicity when dosed correctly [46]. Nitrogen was applied at an initial dose of 20 kg N ha^−1^ before sowing in the form of ammonium nitrate (NH_4_NO_3_), and then supplemented in spring to a total dose of 80 kg N ha^−1^: 40 kg N ha^−1^ in the tillering phase (BBCH 22–24), taking into account nitrogen from ammonium sulphate, and 20 kg N ha^−1^ in the stem elongation phase (BBCH 31–34). Phosphorus and potassium were applied before sowing in amounts of 60 kg P_2_O_5_ ha^−1^ and 80 kg K_2_O ha^−1^, respectively. Meteorological data and used pesticides during the growing season were characterised in a previously published article on this experiment [84].

After harvesting, representative samples of plant material (grain and straw) were taken for laboratory analysis in accordance with the applicable analytical procedures. Yield of plants and content of total, mineral and organic nitrogen are given in a previously published papers [34,37].

### 3.2. Analytical and Statistical Methods

During harvesting, plant material covering an area of 0.25 m^2^ was collected from each experimental unit for laboratory analysis. After separating the grain and straw fractions, the samples were dried at 60 °C until a constant weight was achieved, and then ground in an electric grinder to obtain a homogeneous homogenate. The resulting material was used as a substrate for determining the content of selected elements. The total sulphur content (S_tot_) was determined by the nephelometric method [95], after prior digestion of the samples with fuming nitric acid (V) (HNO_3_). The sulphate sulphur (SO_4_^2−^-S) content was also determined nephelometrically, after extraction of plant material with a 2% acetic acid (CH_3_COOH) solution, according to the methodology of Kurmanbayeva et al. [96]. Additionally, the crushed samples (both grain and straw) were digested in concentrated nitric acid (V), and the selenium (Se) content was determined in the resulting solution using inductively coupled plasma mass spectrometry (ICP-MS) [97], which ensures high sensitivity and precision of elemental analysis. In ICP-MS analysis, the determination range for selenium was 0.005–5 mg·L^−1^, with a limit of detection (LOD) of 0.001 mg·L^−1^. The limits of quantification (LOQ) correspond to the lower limits of the calibration ranges presented above. To ensure the reliability of the results, certified reference materials NCS ZC 73030 (Chinese National Analysis Centre for Iron and Steel, Beijing, China), quality control through blanks, internal standards, multi-point calibration and sensitivity drift checks using control samples were used. In colorimetric and nephelometric methods, calibration curves based on 5–7 points with linearity assessment (R^2^ ≥ 0.99) were prepared, and 3 analytical replicates were performed for each sample to assess precision. Such procedures ensure compliance with validation requirements and minimise the risk of systematic and random errors.

The collected empirical data were subjected to statistical analysis using three-factor analysis of variance (ANOVA), performed in the Statistica computing environment [98,99]. In order to identify significant differences between the mean values of the studied characteristics, Tukey’s multiple post hoc comparison test (HSD) was used, with a significance level of *p* = 0.05 [98]. Based on the test results, the least significant difference (LSD) was determined and statistically homogeneous groups of objects were identified [99]. The values presented in the tables for the interactions of factors A × B × C, which are marked with the same letter, do not show any significant statistical differences. This confirms that they belong to the same homogeneous group within used comparative test. The consistency of the letter designations indicates that the hypothesis of equality of means in the analysed factor combinations is not rejected, in accordance with the criteria adopted in the analysis of variance and post-hoc procedures (Tukey’s test), which allows them to be interpreted as statistically equivalent in the context of the studied feature. The use of the LSD (Least Significant Difference) test in parallel with Tukey’s HSD test stems from the need to balance sensitivity and Type I error control. The LSD test is more liberal and allows for the detection of subtle differences between means, which is useful in preliminary analysis, while Tukey HSD is more conservative and controls for cumulative error in multiple comparisons, ensuring greater reliability of results. In research practice, LSD can indicate potential differences, and Tukey HSD can confirm them, which is particularly important in agronomic studies with multiple factor combinations [100]. Data from individual years were accepted for statistical calculations as repetitions, i.e., statistical calculations were performed for the averages from the years.

## 4. Conclusions

Selenium fertilisation significantly increased the content of this element in spelt wheat and common wheat grains—fourfold and fivefold, respectively, compared to the control. This effect was further modulated by the timing of selenium application to the plant growth environment, indicating the importance of the developmental stage in the biofortification process. On the other hand, the selenium dose and the timing of its application did not show a clear effect on the content of both analysed forms of sulphur, which suggests the complexity of the interaction between these elements. It is important to note that the fertilisation levels did not lead to selenium content considered toxic to humans and animals, confirming the safety of the proposed strategy.

The presence of sulphur in the growing environment of spelt wheat and common wheat was associated with an increase in the total sulphur and sulphate sulphur content in the grain, and especially in the straw. Fertilisation with a higher dose of sulphur increased the total sulphur, sulphate sulphur and organic sulphur content in spelt wheat straw by an average of 17%, 29% and 23%, respectively, and in common wheat straw by 26%, 18% and 57%, respectively. At the same time, the presence of sulphur in the plant growth environment did not affect the selenium content in the grain, which indicates a lack of direct competition in the mechanisms of uptake of these elements under the tested conditions. The results suggest that the optimal dose of selenium for biofortification purposes is 20 mg Se ha^−1^ on clay soil, applied during the stem elongation stage (BBCH 31–34).

Biofortification of wheat through integrated selenium and sulphur fertilisation is an effective agronomic strategy aimed at increasing the selenium content in grain, which in turn may contribute to reducing selenium deficiencies in the human diet. This method is in line with the concept of improving food quality at source, enabling the supply of selenium in an organic form, characterised by high bioavailability and safety for the consumer. In the face of global selenium deficits resulting from its low concentration in the soils of many regions of the world, cereal biofortification appears to be a solution of strategic nutritional and health importance, supporting the prevention of diseases associated with selenium deficiency.

## Figures and Tables

**Table 1:** (a). Content of selenium (mg kg^−1^ d.m.) of grain of spelt wheat and common wheat. (b). Content of selenium (mg kg^−1^ d.m.) of grain of spelt wheat and common wheat, averages for years.

**(a)**																
**Selenium Dose (B)**	**Sulphur Dose (A)**												**Average**			
	**S0**				**SI**				**SII**							
	**I Year**	**II Year**	**III Year**		**I Year**	**II Year**	**III Year**		**I Year**	**II Year**		**III Year**	**I Year**	**II Year**		**III Year**
Spelt wheat																
1st selenium application date (C)																
Se0	0.02	0.02	0.06		0.07	0.03	0.06		0.03	0.04		0.04	0.04	0.03		0.05
SeI	0.11	0.06	0.07		0.08	0.06	0.10		0.06	0.06		0.12	0.08	0.06		0.10
SeII	0.13	0.09	0.06		0.10	0.06	0.10		0.09	0.08		0.03	0.11	0.08		0.06
Average	0.09	0.06	0.06		0.08	0.05	0.09		0.06	0.06		0.06	0.08	0.06		0.07
*r*	0.939	0.997	0.000		0.982	0.866	0.866		1.000	1.000		−0.101	0.997	0.993		0.189
2nd selenium application date (C)																
Se0	0.02	0.02	0.06		0.07	0.03	0.06		0.03	0.04		0.04	0.04	0.03		0.05
SeI	0.15	0.11	0.16		0.13	0.12	0.27		0.17	0.11		0.24	0.15	0.11		0.22
SeII	0.27	0.12	0.34		0.33	0.12	0.25		0.26	0.11		0.38	0.29	0.12		0.32
Average	0.15	0.08	0.19		0.18	0.09	0.19		0.15	0.09		0.22	0.16	0.09		0.20
*r*	1.000	0.908	0.987		0.955	0.866	0.820		0.992	0.866		0.995	0.998	0.912		0.989
LSD_0.05_	A—n.s., B—0.03, C—0.02, A × B—n.s., A × C—n.s., B × C—0.04, A × B × C—n.s.															
Common wheat (*Triticum aestivum* L.)																
1st selenium application date (C)																
Se0	0.02	0.01	0.08		0.02	0.02	0.02		0.02	0.01		0.03	0.02	0.01		0.04
SeI	0.08	0.06	0.19		0.04	0.08	0.10		0.02	0.03		0.13	0.05	0.06		0.14
SeII	0.10	0.09	0.08		0.09	0.09	0.11		0.02	0.05		0.17	0.07	0.08		0.12
Average	0.07	0.05	0.12		0.05	0.06	0.08		0.02	0.03		0.11	0.05	0.05		0.10
*r*	0.961	0.990	0.000		0.971	0.924	0.912		-	1.000		0.971	0.993	0.971		0.756
2nd selenium application date (C)																
Se0	0.02	0.01	0.08		0.02	0.02	0.02		0.02	0.01		0.03	0.02	0.01		0.04
SeI	0.24	0.14	0.14		0.12	0.14	0.21		0.13	0.12		0.20	0.16	0.13		0.18
SeII	0.30	0.22	0.29		0.17	0.17	0.26		0.17	0.22		0.28	0.21	0.20		0.28
Average	0.19	0.12	0.17		0.10	0.11	0.16		0.11	0.12		0.17	0.13	0.12		0.17
*r*	0.950	0.991	0.971		0.982	0.945	0.948		0.966	1.000		0.979	0.965	0.989		0.995
LSD_0.05_	A—0.02, B—0.02, C—0.01, A × B—n.s., A × C—n.s., B × C—0.04, A × B × C—n.s.															
**(b)**																
**Selenium Dose (B)**	**Sulphur Dose (A)**										**Average**				***r***	
	**S0**			**SI**				**SII**								
Spelt wheat																
1st selenium application date (C)																
Se0	0.03 ^a^			0.05 ^ab^				0.04 ^a^			0.04				0.500	
SeI	0.08 ^abc^			0.08 ^abcd^				0.08 ^abc^			0.08				-	
SeII	0.09 ^abcd^			0.09 ^abcd^				0.07 ^ab^			0.08				−0.866	
Average	0.07			0.07				0.06			0.07				−0.866	
*r*	0.933			0.961				0.721			0.866					
2nd selenium application date (C)																
Se0	0.03 ^a^			0.05 ^ab^				0.04 ^a^			0.04				0.500	
SeI	0.14 ^bcde^			0.17 ^cdef^				0.17 ^def^			0.16				0.866	
SeII	0.24 ^ef^			0.23 ^ef^				0.25 ^f^			0.24				0.500	
Average	0.14			0.15				0.15			0.15				0.866	
*r*	1.000			0.982				0.991			0.993					
Average																
Se0	0.03			0.05				0.04			0.04				0.500	
SeI	0.11			0.13				0.13			0.12				0.866	
SeII	0.17			0.16				0.16			0.16				−0.866	
Average	0.11			0.11				0.11			0.11				0.866	
*r*	0.997			0.967				0.961			0.982					
LSD_0.05_	A—n.s., B—0.03, C—0.02, A × B—n.s., A × C—n.s., B × C—0.04, A × B × C—n.s.															
Common wheat (*Triticum aestivum* L.)																
1st selenium application date (C)																
Se0	0.04 ^ab^			0.02 ^a^				0.02 ^a^			0.03				−0.866	
SeI	0.11 ^bcde^			0.07 ^abc^				0.06 ^abc^			0.08				−0.945	
SeII	0.09 ^abc^			0.10 ^abcd^				0.08 ^bcde^			0.09				−0.500	
Average	0.08			0.06				0.05			0.07				−0.982	
*r*	0.693			0.990				0.982			0.933					
2nd selenium application date (C)																
Se0	0.04 ^ab^			0.02 ^a^				0.02 ^a^			0.03				−0.866	
SeI	0.17 ^ef^			0.16 ^cdef^				0.15 ^cdef^			0.16				−1.000	
SeII	0.27 ^g^			0.20 ^def^				0.22 ^fg^			0.23				−0.693	
Average	0.16			0.13				0.13			0.14				−0.866	
*r*	0.997			0.952				0.985			0.985					
Average																
Se0	0.04			0.02				0.02			0.03				−0.866	
SeI	0.14			0.12				0.11			0.12				−0.982	
SeII	0.18			0.15				0.15			0.16				−0.866	
Average	0.12			0.10				0.09			0.11				−0.982	
*r*	0.971			0.955				0.976			0.976					
LSD_0.05_	A—0.02, B—0.02, C—0.01, A × B—n.s., A × C—n.s., B × C—0.04, A × B × C—n.s.															

*r*—correlation coefficient; LSD for A—sulphur dose, B—selenium dose, C—selenium application date; A × B, A × C, B × C, A × B × C—interactions; significant differences at *p* ≤ 0.05; n.s.—differences not significant. Values marked with the same letters do not differ significantly (*p* ≤ 0.05).

**Table 2:** (a). Content of selenium (mg kg^−1^ d.m.) of straw of spelt wheat and common wheat. (b). Content of selenium (mg kg^−1^ d.m.) of straw of spelt wheat and common wheat, averages for years.

**(a)**															
**Selenium Dose (B)**	**Sulphur Dose (A)**											**Average**			
	**S0**				**SI**			**SII**							
	**I Year**	**II Year**	**III Year**		**I Year**	**II Year**	**III Year**	**I Year**	**II Year**		**III Year**	**I Year**		**II Year**	**III Year**
Spelt wheat															
1st selenium application date (C)															
Se0	0.01	0.01	0.01		0.02	0.01	0.01	0.02	0.01		0.01	0.02		0.01	0.01
SeI	0.02	0.01	0.02		0.02	0.01	0.01	0.02	0.01		0.01	0.02		0.01	0.01
SeII	0.02	0.01	0.02		0.03	0.01	0.01	0.03	0.01		0.02	0.03		0.01	0.02
Average	0.02	0.01	0.02		0.02	0.01	0.01	0.02	0.01		0.01	0.02		0.01	0.01
*r*	0.866	-	0.866		0.866	-	-	0.866	-		0.866	0.866		-	0.866
2nd selenium application date (C)															
Se0	0.01	0.01	0.01		0.02	0.01	0.01	0.02	0.01		0.01	0.02		0.01	0.01
SeI	0.02	0.01	0.03		0.03	0.01	0.02	0.03	0.01		0.02	0.03		0.01	0.02
SeII	0.03	0.01	0.03		0.04	0.01	0.02	0.04	0.01		0.03	0.04		0.01	0.03
Average	0.02	0.01	0.02		0.03	0.01	0.02	0.03	0.01		0.02	0.03		0.01	0.02
*r*	1.000	-	0.866		1.000	-	0.866	1.000	-		1.000	1.000		-	1.000
LSD_0.05_	A—n.s., B—n.s., C—n.s., A × B—0.03 A × C—n.s., B × C—n.s., A × B × C—n.s.														
Common wheat															
1st selenium application date (C)															
Se0	0.02	0.01	0.01		0.01	0.01	0.01	0.02	0.01		0.01	0.02		0.01	0.01
SeI	0.01	0.01	0.01		0.02	0.01	0.02	0.02	0.01		0.01	0.02		0.01	0.01
SeII	0.02	0.01	0.01		0.02	0.01	0.02	0.02	0.01		0.01	0.02		0.01	0.01
Average	0.02	0.01	0.01		0.02	0.01	0.02	0.02	0.01		0.01	0.02		0.01	0.01
*r*	0.000	-	-		0.866	-	0.866	-	-		-	-		-	-
2nd selenium application date (C)															
Se0	0.02	0.01	0.01		0.02	0.01	0.01	0.02	0.01		0.01	0.02		0.01	0.01
SeI	0.02	0.01	0.02		0.02	0.01	0.02	0.03	0.01		0.02	0.02		0.01	0.02
SeII	0.03	0.01	0.02		0.03	0.01	0.02	0.03	0.01		0.01	0.03		0.01	0.02
Average	0.02	0.01	0.02		0.02	0.01	0.02	0.03	0.01		0.01	0.02		0.01	0.02
*r*	0.866	-	0.866		0.866	-	0.866	0.866	-		0.000	0.866		-	0.866
LSD_0.05_	A—n.s., B—0.01., C—n.s., A × B—n.s., A × C—n.s., B × C—0.03, A × B × C—n.s.														
**(b)**															
**Selenium Dose (B)**	**Sulphur Dose (A)**									**Average**			***r***		
	**S0**			**SI**			**SII**								
Spelt wheat															
1st selenium application date (C)															
Se0	0.01 ^ab^			0.01 ^a^			0.01 ^a^			0.01			-		
SeI	0.02 ^a^			0.01 ^a^			0.01 ^a^			0.01			−0.866		
SeII	0.02 ^a^			0.02 ^a^			0.02 ^a^			0.02			-		
Average	0.02			0.01			0.01			0.01			−0.866		
*r*	0.866			0.866			0.866			0.866					
2nd selenium application date (C)															
Se0	0.01 ^ab^			0.01 ^a^			0.01 ^a^			0.01			-		
SeI	0.02 ^a^			0.02 ^a^			0.02 ^ab^			0.02			-		
SeII	0.02 ^a^			0.02 ^a^			0.03 ^a^			0.02			0.866		
Average	0.02			0.02			0.02			0.02			-		
*r*	0.866			0.866			1.000			0.866					
Average															
Se0	0.01			0.01			0.01			0.01			-		
SeI	0.02			0.02			0.02			0.02			-		
SeII	0.02			0.02			0.03			0.02			0.866		
Average	0.02			0.02			0.02			0.02			-		
*r*	0.866			0.866			1.000			0.866					
LSD_0.05_	A—n.s., B—n.s., C—n.s., A × B—0.03 A × C—n.s., B × C—n.s., A × B × C—n.s.														
Common wheat															
1st selenium application date (C)															
Se0	0.01 ^a^			0.01 ^a^			0.01 ^a^			0.01			-		
SeI	0.01 ^a^			0.02 ^a^			0.01 ^a^			0.01			0.000		
SeII	0.01 ^a^			0.02 ^a^			0.01 ^a^			0.01			0.000		
Average	0.01			0.02			0.01			0.01			0.000		
*r*	-			0.866			-			-					
2nd selenium application date (C)															
Se0	0.01 ^a^			0.01 ^a^			0.01 ^a^			0.01			-		
SeI	0.02 ^a^			0.02 ^a^			0.02 ^a^			0.02			-		
SeII	0.02 ^a^			0.02 ^b^			0.02 ^a^			0.02			-		
Average	0.02			0.02			0.02			0.02			-		
*r*	0.866			0.866			0.866			0.866					
Average															
Se0	0.01			0.01			0.01			0.01			-		
SeI	0.02			0.02			0.02			0.02			-		
SeII	0.02			0.02			0.02			0.02			-		
Average	0.02			0.02			0.02			0.02			-		
*r*	0.866			0.866			0.866			0.866					
LSD_0.05_	A—n.s., B—0.01., C—n.s., A × B—n.s., A × C—n.s., B × C—0.03, A × B × C—n.s.														

*r*—correlation coefficient; LSD for A—sulphur dose, B—selenium dose, C—selenium application date; A × B, A × C, B × C, A × B × C—interactions; significant differences at *p* ≤ 0.05; n.s.—differences not significant. Values marked with the same letters do not differ significantly (*p* ≤ 0.05).

**Table 3:** (a). Content of total sulphur (g kg^−1^ d.m.) of grain of spelt wheat and common wheat. (b). Content of total sulphur (g kg^−1^ d.m.) of grain of spelt wheat and common wheat, averages for years.

**(a)**															
**Selenium Dose (B)**	**Sulphur Dose (A)**												**Average**		
	**S0**				**SI**				**SII**						
	**I Year**	**II Year**	**III Year**		**I Year**	**II Year**		**III Year**	**I Year**	**II Year**		**III Year**	**I Year**	**II Year**	**III Year**
Spelt wheat															
1st selenium application date (C)															
Se0	1.20	1.16	1.11		1.33	0.98		1.32	1.39	1.02		1.21	1.31	1.05	1.21
SeI	1.74	0.91	1.35		1.61	1.07		1.44	1.58	1.15		1.27	1.64	1.04	1.35
SeII	1.21	0.96	1.10		1.40	1.00		1.19	1.40	0.94		1.10	1.34	0.97	1.13
Average	1.38	1.01	1.19		1.45	1.02		1.32	1.46	1.04		1.19	1.43	1.02	1.23
*r*	0.016	−0.756	−0.035		0.240	0.212		−0.520	0.047	−0.377		−0.638	0.082	−0.918	−0.359
2nd selenium application date (C)															
Se0	1.20	1.16	1.11		1.43	0.98		1.22	1.39	1.02		1.21	1.34	1.05	1.18
SeI	1.28	0.99	1.27		1.34	1.00		1.10	1.43	1.12		1.20	1.35	1.04	1.19
SeII	1.05	0.91	1.00		1.36	0.94		1.00	0.98	0.92		0.81	1.13	0.92	0.94
Average	1.18	1.02	1.13		1.38	0.97		1.11	1.27	1.02		1.07	1.27	1.00	1.10
*r*	−0.642	−0.979	−0.405		−0.741	−0.655		−0.999	−0.823	−0.500		−0.877	−0.845	−0.899	−0.848
LSD_0.05_	A—n.s., B—0.11, C—n.s., A × B—n.s., A × C—n.s., B × C—n.s., A × B × C—n.s.														
Common wheat															
1st selenium application date (C)															
Se0	1.11	0.93	0.99		1.25	0.92		1.25	0.94	0.89		0.99	1.10	0.91	1.08
SeI	1.31	0.86	1.15		1.28	0.91		1.17	1.43	0.93		1.20	1.34	0.90	1.17
SeII	1.45	1.00	1.15		1.44	1.30		1.32	1.14	1.11		1.15	1.34	1.14	1.21
Average	1.29	0.93	1.10		1.32	1.04		1.25	1.17	0.98		1.11	1.26	0.98	1.15
*r*	0.995	0.500	0.866		0.930	0.855		0.466	0.406	0.939		0.729	0.866	0.847	0.976
2nd selenium application date (C)															
Se0	1.11	0.93	0.99		1.25	0.92		1.25	0.94	0.89		0.99	1.10	0.91	1.08
SeI	1.31	1.01	1.29		1.35	1.08		1.21	1.38	1.15		1.29	1.35	1.08	1.26
SeII	1.30	1.11	1.23		1.17	0.98		1.13	1.33	1.1		1.12	1.27	1.06	1.16
Average	1.24	1.02	1.17		1.26	0.99		1.20	1.22	1.05		1.13	1.24	1.02	1.17
*r*	0.843	0.998	0.756		−0.444	0.371		−0.982	0.809	0.761		0.432	0.666	0.807	0.444
LSD_0.05_	A—n.s., B—0.09, C—n.s., A × B—0.20, A × C—n.s., B × C—1.34, A × B × C—n.s.														
**(b)**															
**Selenium Dose (B)**	**Sulphur Dose (A)**										**Average**			***r***	
	**S0**			**SI**			**SII**								
Spelt wheat															
1st selenium application date (C)															
Se0	1.16 ^ab^			1.21 ^ab^			1.21 ^ab^				1.19			0.866	
SeI	1.33 ^ab^			1.37 ^b^			1.33 ^ab^				1.35			0.000	
SeII	1.09 ^ab^			1.20 ^ab^			1.15 ^ab^				1.14			0.545	
Average	1.19			1.26			1.23				1.23			0.569	
*r*	−0.284			−0.052			−0.327				−0.228				
2nd selenium application date (C)															
Se0	1.16 ab			1.21 ab			1.21 ab				1.19			0.866	
SeI	1.18 ab			1.15 ab			1.25 ab				1.19			0.682	
SeII	0.99 a			1.10 ab			0.90 ab				1.00			−0.449	
Average	1.11			1.15			1.12				1.13			0.240	
*r*	−0.814			−0.999			−0.809				−0.866				
Average															
Se0	1.16			1.21			1.21				1.19			0.866	
SeI	1.26			1.26			1.29				1.27			0.866	
SeII	1.04			1.15			1.03				1.07			−0.075	
Average	1.15			1.21			1.18				1.18			0.500	
*r*	−0.545			−0.545			−0.676				−0.596				
LSD_0.05_	A—n.s., B—0.11, C—n.s., A × B—n.s., A × C—n.s., B × C—n.s., A × B × C—n.s.														
Common wheat															
1st selenium application date (C)															
Se0	1.01 ^ab^			1.14 ^ab^			0.94 ^a^				1.03			−0.345	
SeI	1.11 ^ab^			1.12 ^ab^			1.19 ^ab^				1.14			0.918	
SeII	1.20 ^ab^			1.35 ^ab^			1.13 ^ab^				1.23			−0.311	
Average	1.11			1.20			1.09				1.13			−0.171	
*r*	1.000			0.824			0.728				0.998				
2nd selenium application date (C)															
Se0	1.01 ab			1.14 ab			0.94 a				1.03			−0.345	
SeI	1.20 ab			1.21 ab			1.27 ab				1.23			0.924	
SeII	1.21 ab			1.09 ab			1.18 b				1.16			−0.240	
Average	1.14			1.15			1.13				1.14			−0.500	
*r*	0.887			−0.415			0.703				0.640				
Average															
Se0	1.01			1.14			0.94				1.03			−0.345	
SeI	1.16			1.17			1.23				1.19			0.924	
SeII	1.21			1.22			1.16				1.20			−0.778	
Average	1.13			1.18			1.11				1.14			−0.277	
*r*	0.961			0.990			0.727				0.891				
LSD_0.05_	A—n.s., B—0.09, C—n.s., A × B—0.20, A × C—n.s., B × C—1.34, A × B × C—n.s.														

*r*—correlation coefficient; LSD for A—sulphur dose, B—selenium dose, C—selenium application date; A × B, A × C, B × C, A × B × C—interactions; significant differences at *p* ≤ 0.05; n.s.—differences not significant. Values marked with the same letters do not differ significantly (*p* ≤ 0.05).

**Table 4:** (a). Content of total sulphur (g kg^−1^ d.m.) of straw of spelt wheat and common wheat. (b). Content of total sulphur (g kg^−1^ d.m.) of straw of spelt wheat and common wheat, averages for years.

**(a)**															
**Selenium Dose (B)**	**Sulphur Dose (A)**												**Average**		
	**S0**				**SI**				**SII**						
	**I Year**	**II Year**	**III Year**		**I Year**	**II Year**		**III Year**	**I Year**	**II Year**		**III Year**	**I Year**	**II Year**	**III Year**
Spelt wheat															
1st selenium application date (C)															
Se0	0.25	0.53	0.33		0.17	0.45		0.37	0.31	0.64		0.48	0.24	0.54	0.39
SeI	0.32	0.38	0.26		0.50	0.43		0.29	0.34	0.58		0.37	0.39	0.46	0.31
SeII	0.31	0.44	0.31		0.27	0.29		0.19	0.09	0.48		0.54	0.22	0.40	0.35
Average	0.29	0.45	0.30		0.31	0.39		0.28	0.25	0.57		0.46	0.28	0.47	0.35
*r*	0.792	−0.596	−0.277		0.295	−0.918		−0.998	−0.806	−0.990		0.348	−0.108	−0.997	−0.500
2nd selenium application date (C)															
Se0	0.25	0.53	0.33		0.17	0.45		0.37	0.31	0.64		0.48	0.24	0.54	0.39
SeI	0.22	0.55	0.29		0.29	0.39		0.28	0.19	0.45		0.37	0.23	0.46	0.31
SeII	0.30	0.33	0.31		0.43	0.28		0.28	0.28	0.47		0.31	0.34	0.36	0.30
Average	0.26	0.47	0.31		0.30	0.37		0.31	0.26	0.52		0.39	0.27	0.45	0.34
*r*	0.619	−0.822	−0.500		0.999	−0.986		−0.866	−0.240	−0.814		−0.986	0.822	−0.998	−0.912
LSD_0.05_	A—0.05, B—n.s., C—n.s., A × B—n.s., A × C—n.s., B × C—n.s., A × B × C—n.s.														
Common wheat															
1st selenium application date (C)															
Se0	0.13	0.26	0.18		0.29	0.27		0.28	0.33	0.44		0.28	0.25	0.27	0.25
SeI	0.13	0.20	0.14		0.14	0.23		0.18	0.16	0.21		0.18	0.14	0.21	0.17
SeII	0.22	0.26	0.23		0.20	0.15		0.17	0.59	0.40		0.48	0.28	0.27	0.28
Average	0.14	0.24	0.18		0.17	0.22		0.20	0.36	0.29		0.31	0.22	0.25	0.23
*r*	0.866	0.000	0.554		−0.596	−0.982		−0.904	0.600	−0.163		0.655	0.203	0.000	0.264
2nd selenium application date (C)															
Se0	0.13	0.26	0.18		0.29	0.27		0.28	0.33	0.44		0.28	0.25	0.27	0.25
SeI	0.13	0.28	0.16		0.18	0.23		0.21	0.30	0.25		0.30	0.17	0.25	0.21
SeII	0.29	0.62	0.42		0.27	0.34		0.33	0.15	0.20		0.18	0.24	0.39	0.31
Average	0.15	0.39	0.24		0.25	0.28		0.27	0.26	0.24		0.25	0.22	0.30	0.26
*r*	0.866	0.890	0.829		−0.171	0.629		0.415	−0.933	−0.948		−0.778	−0.115	0.792	0.596
LSD_0.05_	A—0.05, B—0.05, C—n.s., A × B—0.12, A × C—0.08, B × C—n.s., A × B × C—0.17														
**(b)**															
**Selenium Dose (B)**	**Sulphur Dose (A)**										**Average**			***r***	
	**S0**			**SI**			**SII**								
Spelt wheat															
1st selenium application date (C)															
Se0	0.37 ^ab^			0.33 ^ab^			0.48 ^b^				0.39			0.708	
SeI	0.32 ^ab^			0.41 ^ab^			0.43 ^ab^				0.39			0.939	
SeII	0.35 ^ab^			0.25 ^a^			0.37 ^ab^				0.32			0.156	
Average	0.35			0.33			0.43				0.37			0.756	
*r*	−0.397			−0.500			−0.999				−0.866				
2nd selenium application date (C)															
Se0	0.37 ^ab^			0.33 ^ab^			0.48 ^b^				0.39			0.708	
SeI	0.35 ^ab^			0.32 ^ab^			0.34 ^ab^				0.34			−0.327	
SeII	0.31 ^ab^			0.33 ^ab^			0.35 ^ab^				0.33			1.000	
Average	0.35			0.33			0.39				0.35			0.655	
*r*	−0.982			0.000			−0.832				−0.933				
Average															
Se0	0.37			0.33			0.48				0.39			0.708	
SeI	0.34			0.37			0.39				0.37			0.993	
SeII	0.33			0.29			0.36				0.33			0.427	
Average	0.35			0.33			0.41				0.36			0.721	
*r*	−0.961			−0.500			−0.961				−0.982				
LSD_0.05_	A—0.05, B—n.s., C—n.s., A × B—n.s., A × C—n.s., B × C—n.s., A × B × C—n.s.														
Common wheat															
1st selenium application date (C)															
Se0	0.19 ^abc^			0.28 a^bc^			0.29 ^bcd^				0.25			0.908	
SeI	0.16 ^abc^			0.18 ^abc^			0.18 ^abc^				0.17			0.866	
SeII	0.22 ^abc^			0.12 ^ab^			0.49 ^e^				0.28			0.705	
Average	0.19			0.19			0.32				0.24			0.866	
*r*	0.500			−0.990			0.636				0.264				
2nd selenium application date (C)															
Se0	0.19 ^abc^			0.28 ^abc^			0.29 ^bcd^				0.25			0.908	
SeI	0.14 ^a^			0.21 ^abc^			0.28 ^abcd^				0.21			1.000	
SeII	0.44 ^de^			0.31 ^cd^			0.18 ^abc^				0.31			−1.000	
Average	0.26			0.27			0.25				0.26			−0.500	
*r*	0.778			0.292			−0.904				0.596				
Average															
Se0	0.19			0.28			0.29				0.25			0.908	
SeI	0.15			0.20			0.23				0.19			0.990	
SeII	0.33			0.22			0.34				0.30			0.075	
Average	0.23			0.23			0.29				0.25			0.866	
*r*	0.741			−0.721			0.454				0.454				
LSD_0.05_	A—0.05, B—0.05, C—n.s., A × B—0.12, A × C—0.08, B × C—n.s., A × B × C—0.17														

*r*—correlation coefficient; LSD for A—sulphur dose, B—selenium dose, C—selenium application date; A × B, A × C, B × C, A × B × C—interactions; significant differences at *p* ≤ 0.05; n.s.—differences not significant. Values marked with the same letters do not differ significantly (*p* ≤ 0.05).

**Table 5:** (a). Content of sulphate sulphur (g kg^−1^ d.m.) of grain of spelt wheat and common wheat. (b). Content of sulphate sulphur (g kg^−1^ d.m.) of grain of spelt wheat and common wheat, averages for years.

**(a)**																
**Selenium Dose (B)**	**Sulphur Dose (A)**												**Average**			
	**S0**				**SI**				**SII**							
	**I Year**	**II Year**	**III Year**		**I Year**	**II Year**		**III Year**	**I Year**	**II Year**		**III Year**	**I Year**		**II Year**	**III Year**
Spelt wheat																
1st selenium application date (C)																
Se0	0.33	0.28	0.20		0.35	0.28		0.36	0.35	0.50		0.48	0.34		0.35	0.35
SeI	0.43	0.26	0.16		0.36	0.33		0.55	0.28	0.58		0.49	0.36		0.39	0.40
SeII	0.22	0.26	0.10		0.50	0.17		0.45	0.41	0.31		0.35	0.38		0.25	0.30
Average	0.33	0.27	0.15		0.40	0.26		0.45	0.35	0.46		0.44	0.36		0.33	0.35
*r*	−0.524	−0.866	−0.993		0.894	−0.672		0.473	0.461	−0.685		−0.832	1.000		−0.693	−0.500
2nd selenium application date (C)																
Se0	0.33	0.28	0.20		0.35	0.28		0.36	0.35	0.50		0.48	0.34		0.35	0.35
SeI	0.45	0.24	0.16		0.39	0.13		0.29	0.32	0.21		0.50	0.39		0.19	0.32
SeII	0.43	0.26	0.27		0.37	0.44		0.21	0.40	0.32		0.73	0.40		0.34	0.40
Average	0.40	0.26	0.21		0.37	0.28		0.29	0.36	0.34		0.57	0.38		0.30	0.36
*r*	0.778	−0.500	0.629		0.500	0.516		−0.999	0.619	−0.615		0.900	0.933		−0.056	0.619
LSD_0.05_	A—0.06, B—n.s., C—n.s., A × B—n.s., A × C—n.s., B × C—0.10, A × B × C—n.s.															
Common wheat																
1st selenium application date (C)																
Se0	0.42	0.90	0.36		0.61	0.37		0.45	0.63	0.22		0.17	0.55		0.50	0.33
SeI	0.66	0.52	0.30		0.46	0.21		0.36	0.62	0.55		0.71	0.58		0.43	0.46
SeII	0.70	0.51	0.18		0.48	0.32		0.43	0.42	0.28		0.71	0.53		0.37	0.44
Average	0.59	0.64	0.28		0.52	0.30		0.41	0.56	0.35		0.53	0.56		0.43	0.41
*r*	0.924	−0.877	−0.982		−0.798	−0.305		−0.212	−0.886	0.171		0.866	−0.397		−0.999	0.786
2nd selenium application date (C)																
Se0	0.42	0.90	0.36		0.61	0.37		0.45	0.63	0.22		0.17	0.55		0.50	0.33
SeI	0.59	0.68	0.39		0.64	0.22		0.38	0.75	0.34		0.44	0.66		0.41	0.40
SeII	0.58	0.38	0.35		0.72	0.19		0.33	0.52	0.33		0.45	0.61		0.30	0.38
Average	0.53	0.65	0.37		0.66	0.26		0.39	0.63	0.30		0.35	0.61		0.40	0.37
*r*	0.839	−0.996	−0.240		0.967	−0.933		−0.995	−0.478	0.826		0.881	0.545		−0.998	0.693
LSD_0.05_	A—0.08, B—n.s., C—n.s., A × B—0.19, A × C—n.s., B × C—n.s., A × B × C—n.s.															
**(b)**																
**Selenium Dose (B)**	**Sulphur Dose (A)**										**Average**			***r***		
	**S0**			**SI**			**SII**									
Spelt wheat																
1st selenium application date (C)																
Se0	0.27 ^ab^			0.33 ^abc^			0.44 ^bc^				0.35			0.986		
SeI	0.28 ^abc^			0.41 ^bc^			0.45 ^bc^				0.38			0.956		
SeII	0.19 ^a^			0.37 ^abc^			0.36 ^abc^				0.31			0.840		
Average	0.25			0.37			0.42				0.35			0.973		
*r*	−0.811			0.500			−0.811				−0.569					
2nd selenium application date (C)																
Se0	0.27 ^ab^			0.33 ^abc^			0.44 ^bc^				0.35			0.986		
SeI	0.28 ^ab^			0.27 ^ab^			0.34 ^abc^				0.30			0.792		
SeII	0.32 ^abc^			0.34 ^abc^			0.48 c				0.38			0.918		
Average	0.29			0.31			0.42				0.34			0.929		
*r*	0.945			0.132			0.277				0.371					
Average																
Se0	0.27			0.33			0.44				0.35			0.986		
SeI	0.28			0.34			0.40				0.34			1.000		
SeII	0.26			0.36			0.42				0.35			0.990		
Average	0.27			0.34			0.42				0.35			0.999		
*r*	−0.500			0.982			−0.500				0.000					
LSD_0.05_	A—0.06, B—n.s., C—n.s., A × B—n.s., A × C—n.s., B × C—0.10, A × B × C—n.s.															
Common wheat																
1st selenium application date (C)																
Se0	0.56 ^a^			0.48 ^a^			0.34 ^a^				0.46			−0.988		
SeI	0.49 ^a^			0.34 ^a^			0.63 ^a^				0.49			0.483		
SeII	0.46 ^a^			0.41 ^a^			0.47 ^a^				0.45			0.156		
Average	0.51			0.41			0.48				0.46			−0.292		
*r*	−0.974			−0.500			0.447				−0.240					
2nd selenium application date (C)																
Se0	0.56 ^a^			0.48 ^a^			0.34 ^a^				0.46			−0.988		
SeI	0.55 ^a^			0.41 ^a^			0.51 ^a^				0.49			−0.277		
SeII	0.44 ^a^			0.41 ^a^			0.43 ^a^				0.43			−0.327		
Average	0.52			0.43			0.43				0.46			−0.866		
*r*	−0.901			−0.866			0.529				−0.500					
Average																
Se0	0.56			0.48			0.34				0.46			−0.988		
SeI	0.52			0.38			0.57				0.49			0.254		
SeII	0.45			0.41			0.45				0.44			0.000		
Average	0.52			0.42			0.46				0.46			−0.596		
*r*	−0.988			−0.682			0.478				−0.397					
LSD_0.05_	A—0.08, B—n.s., C—n.s., A × B—0.19, A × C—n.s., B × C—n.s., A × B × C—n.s.															

*r*—correlation coefficient; LSD for A—sulphur dose, B—selenium dose, C—selenium application date; A × B, A × C, B × C, A × B × C—interactions; significant differences at *p* ≤ 0.05; n.s.—differences not significant. Values marked with the same letters do not differ significantly (*p* ≤ 0.05).

**Table 6:** (a). Content of sulphate sulphur (g kg^−1^ d.m.) of straw of spelt wheat and common wheat. (b). Content of sulphate sulphur (g kg^−1^ d.m.) of straw of spelt wheat and common wheat, averages for years.

**(a)**																
**Selenium Dose (B)**	**Sulphur Dose (A)**												**Average**			
	**S0**				**SI**				**SII**							
	**I Year**	**II Year**	**III Year**		**I Year**	**II Year**		**III Year**	**I Year**	**II Year**		**III Year**	**I Year**		**II Year**	**III Year**
Spelt wheat																
1st selenium application date (C)																
Se0	0.14	0.13	0.15		0.11	0.19		0.17	0.21	0.22		0.17	0.15		0.18	0.16
SeI	0.18	0.12	0.22		0.13	0.12		0.09	0.20	0.13		0.14	0.17		0.12	0.15
SeII	0.09	0.18	0.07		0.36	0.14		0.07	0.24	0.15		0.12	0.23		0.16	0.09
Average	0.14	0.14	0.15		0.20	0.15		0.11	0.22	0.17		0.14	0.18		0.15	0.13
*r*	−0.554	0.778	−0.533		0.900	−0.693		−0.945	0.721	−0.741		−0.993	0.961		−0.327	−0.924
2nd selenium application date (C)																
Se0	0.14	0.13	0.15		0.11	0.19		0.17	0.21	0.22		0.17	0.15		0.18	0.16
SeI	0.19	0.13	0.10		0.23	0.09		0.07	0.12	0.21		0.08	0.18		0.14	0.08
SeII	0.21	0.17	0.06		0.18	0.14		0.18	0.31	0.14		0.08	0.23		0.15	0.11
Average	0.18	0.14	0.10		0.17	0.14		0.14	0.21	0.19		0.11	0.19		0.16	0.12
*r*	0.971	0.866	−0.998		0.581	−0.500		0.082	0.526	−0.918		−0.866	0.990		−0.721	−0.619
LSD_0.05_	A—0.02, B—n.s., C—n.s., A × B—0.06, A × C—n.s., B × C—n.s., A × B × C—n.s.															
Common wheat																
1st selenium application date (C)																
Se0	0.12	0.23	0.18		0.11	0.20		0.15	0.15	0.27		0.16	0.13		0.29	0.16
SeI	0.12	0.18	0.12		0.13	0.18		0.12	0.18	0.17		0.07	0.14		0.18	0.10
SeII	0.17	0.19	0.20		0.08	0.14		0.13	0.17	0.16		0.18	0.20		0.16	0.18
Average	0.15	0.20	0.17		0.15	0.17		0.14	0.17	0.26		0.14	0.16		0.21	0.15
*r*	0.866	−0.756	0.240		−0.596	−0.982		−0.655	0.655	−0.904		0.171	0.924		−0.929	0.240
2nd selenium application date (C)																
Se0	0.12	0.23	0.18		0.11	0.20		0.15	0.15	0.27		0.16	0.13		0.29	0.16
SeI	0.03	0.17	0.12		0.09	0.20		0.15	0.27	0.15		0.16	0.16		0.17	0.16
SeII	0.26	0.17	0.12		0.16	0.15		0.15	0.15	0.17		0.14	0.19		0.16	0.14
Average	0.17	0.19	0.15		0.12	0.18		0.15	0.19	0.25		0.15	0.16		0.21	0.15
*r*	0.604	−0.866	−0.866		0.693	−0.866		-	0.000	−0.778		−0.866	1.000		−0.899	−0.866
LSD_0.05_	A—0.03, B—0.03, C—n.s., A × B—0.07, A × C—n.s., B × C—n.s., A × B × C—n.s.															
**(b)**																
**Selenium Dose (B)**	**Sulphur Dose (A)**										**Average**			***r***		
	**S0**			**SI**			**SII**									
Spelt wheat																
1st selenium application date (C)																
Se0	0.14 ^a^			0.16 ^a^			0.20 ^a^				0.17			0.982		
SeI	0.17 ^a^			0.11 ^a^			0.16 ^a^				0.15			−0.156		
SeII	0.11 ^a^			0.19 ^a^			0.17 ^a^				0.16			0.721		
Average	0.14			0.15			0.18				0.16			0.961		
*r*	−0.500			0.371			−0.721				−0.500					
2nd selenium application date (C)																
Se0	0.14 ^a^			0.16 ^a^			0.20 ^a^				0.17			0.982		
SeI	0.14 ^a^			0.13 ^a^			0.14 ^a^				0.14			0.000		
SeII	0.15 ^a^			0.17 ^a^			0.18 ^a^				0.16			0.982		
Average	0.14			0.15			0.17				0.15			0.982		
*r*	0.866			0.240			−0.327				−0.327					
Average																
Se0	0.14			0.16			0.20				0.17			0.982		
SeI	0.16			0.12			0.15				0.15			−0.240		
SeII	0.13			0.18			0.18				0.16			0.866		
Average	0.14			0.15			0.18				0.16			0.961		
*r*	−0.327			0.327			−0.397				−0.500					
LSD_0.05_	A—0.02, B—n.s., C—n.s., A × B—0.06, A × C—n.s., B × C—n.s., A × B × C—n.s.															
Common wheat																
1st selenium application date (C)																
Se0	0.18 ^ab^			0.15 ^ab^			0.25 ^b^				0.19			0.682		
SeI	0.14 ^ab^			0.14 ^ab^			0.14 ^a^				0.14			-		
SeII	0.20 ^ab^			0.17 ^ab^			0.17 ^ab^				0.18			−0.866		
Average	0.17			0.15			0.19				0.17			0.500		
*r*	0.327			0.655			−0.703				−0.189					
2nd selenium application date (C)																
Se0	0.18 ^ab^			0.15 ^ab^			0.25 ^b^				0.19			0.682		
SeI	0.15 ^a^			0.15 ^ab^			0.19 ^ab^				0.16			0.866		
SeII	0.18 ^ab^			0.15 ^ab^			0.15 ^ab^				0.16			−0.866		
Average	0.17			0.15			0.20				0.17			0.596		
*r*	0.000			-			−0.993				−0.866					
Average																
Se0	0.18			0.15			0.25				0.19			0.682		
SeI	0.15			0.15			0.17				0.15			0.866		
SeII	0.19			0.16			0.16				0.17			−0.866		
Average	0.17			0.15			0.20				0.17			0.596		
*r*	0.240			0.866			−0.912				−0.500					
LSD_0.05_	A—0.03, B—0.03, C—n.s., A × B—0.07, A × C—n.s., B × C—n.s., A × B × C—n.s.															

*r*—correlation coefficient; LSD for A—sulphur dose, B—selenium dose, C—selenium application date; A × B, A × C, B × C, A × B × C—interactions; significant differences at *p* ≤ 0.05; n.s.—differences not significant. Values marked with the same letters do not differ significantly (*p* ≤ 0.05).

**Table 7:** (a). Content of organic sulphur (g kg^−1^ d.m.) of grain of spelt wheat and common wheat. (b). Content of organic sulphur (g kg^−1^ d.m.) of grain of spelt wheat and common wheat, averages for years.

**(a)**																
**Selenium Dose (B)**	**Sulphur Dose (A)**												**Average**			
	**S0**				**SI**				**SII**							
	**I Year**	**II Year**	**III Year**		**I Year**	**II Year**	**III Year**		**I Year**	**II Year**		**III Year**	**I Year**		**II Year**	**III Year**
Spelt wheat																
1st selenium application date (C)																
Se0	0.87	0.88	0.91		0.98	0.70	0.96		1.04	0.52		0.73	0.97		0.70	0.86
SeI	1.31	0.65	1.19		1.25	0.74	0.89		1.30	0.57		0.78	1.28		0.65	0.95
SeII	0.99	0.70	1.00		0.90	0.83	0.74		0.99	0.63		0.75	0.96		0.72	0.83
Average	1.05	0.74	1.04		1.05	0.76	0.87		1.11	0.58		0.75	1.07		0.69	0.88
*r*	0.264	−0.744	0.315		−0.218	0.976	−0.979		−0.150	0.999		0.397	−0.027		0.277	−0.240
2nd selenium application date (C)																
Se0	0.87	0.88	0.91		1.08	0.70	0.86		1.04	0.52		0.73	1.00		0.70	0.83
SeI	0.83	0.75	1.11		0.95	0.87	0.81		1.11	0.91		0.70	0.96		0.85	0.87
SeII	0.62	0.65	0.73		0.99	0.50	0.79		0.58	0.60		0.08	0.73		0.58	0.54
Average	0.78	0.76	0.92		1.01	0.69	0.82		0.91	0.68		0.50	0.89		0.70	0.74
*r*	−0.931	−0.997	−0.473		−0.676	−0.540	−0.971		−0.799	0.194		−0.886	−0.926		−0.444	−0.805
LSD_0.05_	A—n.s., B—0.11, C—n.s., A × B—n.s., A × C—n.s., B × C—n.s., A × B × C—n.s.															
Common wheat																
1st selenium application date (C)																
Se0	0.69	0.03	0.63		0.64	0.55	0.80		0.31	0.67		0.82	0.55		0.41	0.75
SeI	0.65	0.34	0.85		0.82	0.70	0.81		0.81	0.38		0.49	0.76		0.47	0.71
SeII	0.75	0.49	0.97		0.96	0.98	0.89		0.72	0.83		0.44	0.81		0.77	0.77
Average	0.70	0.29	0.82		0.80	0.74	0.84		0.61	0.63		0.58	0.70		0.55	0.74
*r*	0.596	0.980	0.986		0.997	0.985	0.912		0.769	0.351		−0.920	0.942		0.933	0.327
2nd selenium application date (C)																
Se0	0.69	0.03	0.63		0.64	0.55	0.80		0.31	0.67		0.82	0.55		0.41	0.75
SeI	0.72	0.33	0.90		0.71	0.86	0.83		0.63	0.81		0.85	0.69		0.67	0.86
SeII	0.72	0.73	0.88		0.45	0.79	0.80		0.81	0.77		0.67	0.66		0.76	0.78
Average	0.71	0.37	0.80		0.60	0.73	0.81		0.59	0.75		0.78	0.63		0.62	0.80
*r*	0.866	0.997	0.831		−0.706	0.738	0.000		0.987	0.693		−0.778	0.746		0.963	0.264
LSD_0.05_	A—0.11, B—0.11, C—n.s., A × B—n.s., A × C—n.s., B × C—n.s., A × B × C—n.s.															
**(b)**																
**Selenium Dose (B)**	**Sulphur Dose (A)**										**Average**			***r***		
	**S0**			**SI**				**SII**								
Spelt wheat																
1st selenium application date (C)																
Se0	0.89 ^a^			0.88 ^a^				0.77 ^a^			0.84			−0.901		
SeI	1.05 ^a^			0.96 ^a^				0.88 ^a^			0.97			−0.999		
SeII	0.90 ^a^			0.83 ^a^				0.79 ^a^			0.83			−0.988		
Average	0.94			0.89				0.81			0.88			−0.991		
*r*	0.056			−0.381				0.171			−0.064					
2nd selenium application date (C)																
Se0	0.89 ^a^			0.88 ^a^				0.77 ^a^			0.84			−0.901		
SeI	0.90 ^a^			0.88 ^a^				0.91 ^a^			0.89			0.327		
SeII	0.67 ^a^			0.76 ^a^				0.42 ^a^			0.62			−0.710		
Average	0.82			0.84				0.70			0.79			−0.792		
*r*	−0.846			−0.866				−0.693			−0.766					
Average																
Se0	0.89			0.88				0.77			0.84			−0.901		
SeI	0.98			0.92				0.90			0.93			−0.961		
SeII	0.79			0.80				0.61			0.73			−0.842		
Average	0.88			0.87				0.76			0.84			−0.901		
*r*	−0.526			−0.655				−0.551			−0.549					
LSD_0.05_	A—n.s., B—0.11, C—n.s., A × B—n.s., A × C—n.s., B × C—n.s., A × B × C—n.s.															
Common wheat																
1st selenium application date (C)																
Se0	0.45 ^a^			0.66 ^a^				0.60 ^a^			0.57			0.693		
SeI	0.62 ^a^			0.78 ^a^				0.56 ^a^			0.65			−0.264		
SeII	0.74 ^a^			0.94 ^a^				0.66 ^a^			0.78			−0.277		
*r*	0.995			0.997				0.596			0.991					
Average	0.60			0.79				0.61			0.67			0.047		
2nd selenium application date (C)																
Se0	0.45 ^a^			0.66 ^a^				0.60 ^a^			0.57			0.693		
SeI	0.65 ^a^			0.80 ^a^				0.76 ^a^			0.74			0.708		
SeII	0.77 ^a^			0.68 ^a^				0.75 ^a^			0.73			−0.212		
Average	0.62			0.72				0.70			0.68			0.756		
*r*	0.990			0.132				0.837			0.839					
Average																
Se0	0.45			0.66				0.60			0.57			0.693		
SeI	0.64			0.79				0.66			0.70			0.123		
SeII	0.76			0.81				0.71			0.76			−0.500		
Average	0.61			0.76				0.66			0.68			0.327		
*r*	0.992			0.921				0.999			0.978					
LSD_0.05_	A—0.11, B—0.11, C—n.s., A × B—n.s., A × C—n.s., B × C—n.s., A × B × C—n.s.															

*r*—correlation coefficient; LSD for A—sulphur dose, B—selenium dose, C—selenium application date; A × B, A × C, B × C, A × B × C—interactions; significant differences at *p* ≤ 0.05; n.s.—differences not significant. Values marked with the same letters do not differ significantly (*p* ≤ 0.05).

**Table 8:** (a). Content of organic sulphur (g kg^−1^ d.m.) of straw of spelt wheat and common wheat. (b). Content of organic sulphur (g kg^−1^ d.m.) of straw of spelt wheat and common wheat, averages for years.

**(a)**																
**Selenium Dose (B)**	**Sulphur Dose (A)**												**Average**			
	**S0**				**SI**				**SII**							
	**I Year**	**II Year**	**III Year**		**I Year**	**II Year**	**III Year**		**I Year**	**II Year**		**III Year**	**I Year**		**II Year**	**III Year**
Spelt wheat																
1st selenium application date (C)																
Se0	0.11	0.40	0.18		0.06	0.26	0.20		0.10	0.42		0.31	0.09		0.36	0.23
SeI	0.14	0.26	0.04		0.37	0.31	0.20		0.14	0.45		0.23	0.22		0.34	0.16
SeII	0.22	0.26	0.24		0.09	0.15	0.12		0.15	0.33		0.42	0.01		0.24	0.26
Average	0.15	0.31	0.15		0.11	0.24	0.17		0.03	0.40		0.32	0.10		0.32	0.22
*r*	0.967	−0.866	0.292		0.088	−0.672	−0.866		0.945	−0.721		0.577	−0.377		−0.933	0.292
2nd selenium application date (C)																
Se0	0.11	0.40	0.18		0.06	0.26	0.20		0.10	0.42		0.31	0.09		0.36	0.23
SeI	0.03	0.42	0.19		0.06	0.30	0.21		0.07	0.24		0.29	0.05		0.32	0.23
SeII	0.09	0.16	0.25		0.25	0.14	0.10		0.03	0.33		0.23	0.11		0.21	0.19
Average	0.08	0.33	0.21		0.13	0.23	0.17		0.05	0.33		0.28	0.08		0.29	0.22
*r*	−0.240	−0.829	0.924		0.866	−0.721	−0.822		−0.997	−0.500		−0.961	0.327		−0.966	−0.866
LSD_0.05_	A—n.s., B—n.s., C—n.s., A × B—n.s., A × C—n.s., B × C—n.s., A × B × C—n.s.															
Common wheat																
1st selenium application date (C)																
Se0	0.01	0.03	0.02		0.18	0.07	0.13		0.18	0.17		0.12	0.07		0.02	0.14
SeI	0.01	0.02	0.02		0.01	0.05	0.06		0.02	0.04		0.11	0.02		0.01	0.09
SeII	0.05	0.07	0.03		0.12	0.01	0.04		0.42	0.24		0.30	0.01		0.19	0.08
Average	0.01	0.04	0.02		0.06	0.05	0.07		0.20	0.09		0.17	0.03		0.07	0.10
*r*	0.866	0.756	0.866		−0.348	−0.982	−0.952		0.596	0.345		0.842	−0.933		0.840	−0.933
2nd selenium application date (C)																
Se0	0.01	0.03	0.02		0.18	0.07	0.13		0.18	0.17		0.12	0.12		0.09	0.09
SeI	0.10	0.11	0.04		0.09	0.03	0.06		0.03	0.10		0.14	0.07		0.08	0.08
SeII	0.03	0.45	0.30		0.11	0.19	0.18		0.00	0.03		0.04	0.05		0.22	0.17
Average	0.01	0.20	0.11		0.13	0.10	0.12		0.07	0.05		0.10	0.07		0.11	0.11
*r*	0.212	0.942	0.896		−0.741	0.721	0.415		−0.933	−1.000		−0.756	−0.971		0.832	0.811
LSD_0.05_	A—n.s., B—n.s., C—3.78, A × B—0.13, A × C—0.10, B × C—n.s., A × B × C—0.21															
**(b)**																
**Selenium Dose (B)**	**Sulphur Dose (A)**										**Average**			***r***		
	**S0**			**SI**				**SII**								
Spelt wheat																
1st selenium application date (C)																
Se0	0.23 ^a^			0.17 ^a^				0.28 ^a^			0.22			0.454		
SeI	0.15 ^a^			0.30 ^a^				0.27 ^a^			0.24			0.756		
SeII	0.24 ^a^			0.11 ^a^				0.20 ^a^			0.16			−0.300		
Average	0.21			0.19				0.25			0.21			0.655		
*r*	0.101			−0.309				−0.918			−0.721					
2nd selenium application date (C)																
Se0	0.23 ^a^			0.17 ^a^				0.28 ^a^			0.22			0.454		
SeI	0.21 ^a^			0.19 ^a^				0.20 ^a^			0.20			−0.500		
SeII	0.16 ^a^			0.16 ^a^				0.17 ^a^			0.17			0.866		
Average	0.21			0.18				0.22			0.20			0.240		
*r*	−0.971			−0.327				−0.967			−0.993					
Average																
Se0	0.23			0.17				0.28			0.22			0.454		
SeI	0.18			0.25				0.24			0.22			0.792		
SeII	0.20			0.11				0.19			0.17			−0.101		
Average	0.21			0.19				0.24			0.21			0.596		
*r*	−0.596			−0.427				−0.998			−0.866					
LSD_0.05_	A—n.s., B—n.s., C—n.s., A × B—n.s., A × C—n.s., B × C—n.s., A × B × C—n.s.															
Common wheat																
1st selenium application date (C)																
Se0	0.02 ^a^			0.13 ^abc^				0.16 ^a^			0.08			0.950		
SeI	0.02 ^a^			0.04 ^a^				0.06 ^a^			0.03			1.000		
SeII	0.05 ^a^			0.06 ^a^				0.32 ^c^			0.09			0.882		
Average	0.02			0.06				0.15			0.07			0.976		
*r*	0.866			−0.741				0.610			0.156					
2nd selenium application date (C)																
Se0	0.02 ^a^			0.13 ^abc^				0.16 ^a^			0.10			0.950		
SeI	0.08 ^a^			0.06 ^ab^				0.09 ^ab^			0.08			0.327		
SeII	0.26 ^bc^			0.16 ^abc^				0.02 ^a^			0.15			−0.995		
Average	0.11			0.12				0.07			0.10			−0.756		
*r*	0.961			0.292				−1.000			0.693					
Average																
Se0	0.02			0.13				0.16			0.09			0.950		
SeI	0.05			0.05				0.08			0.06			0.866		
SeII	0.16			0.11				0.17			0.12			0.156		
Average	0.07			0.09				0.11			0.09			1.000		
*r*	0.950			−0.240				0.101			0.500					
LSD_0.05_	A—n.s., B—n.s., C—3.78, A × B—0.13, A × C—0.10, B × C—n.s., A × B × C—0.21															

*r*—correlation coefficient; LSD for A—sulphur dose, B—selenium dose, C—selenium application date; A × B, A × C, B × C, A × B × C—interactions; significant differences at *p* ≤ 0.05; n.s.—differences not significant. Values marked with the same letters do not differ significantly (*p* ≤ 0.05).

**Table 9:** (a). N:S ratio in grain of spelt wheat and common wheat. (b). N:S ratio in grain of spelt wheat and common wheat, averages for years.

**(a)**																
**Selenium Dose (B)**	**Sulphur Dose (A)**												**Average**			
	**S0**				**SI**				**SII**							
	**I Year**	**II Year**	**III Year**		**I Year**	**II Year**	**III Year**		**I Year**	**II Year**		**III Year**	**I Year**		**II Year**	**III Year**
Spelt wheat																
1st selenium application date (C)																
Se0	16.88	17.14	20.43		17.20	22.09	16.90		15.11	18.90		16.66	16.31		19.02	17.95
SeI	12.44	19.38	16.24a		13.16	17.01	16.27		12.99	16.63		17.27	12.88		17.62	16.61
SeII	17.74	18.86	19.26		14.86	18.39	18.28		14.06	17.67		19.17	15.42		18.25	18.88
Average	15.30	18.36	18.43		14.91	19.03	17.05		13.98	17.35		17.70	14.72		18.30	17.75
*r*	0.151	0.734	−0.271		−0.577	−0.704	0.671		−0.495	−0.541		0.959	−0.250		−0.549	0.407
2nd selenium application date (C)																
Se0	16.88	17.14	20.43		15.99	22.09	18.29		15.11	18.03		16.66	16.31		19.02	18.41
SeI	15.68	17.91	16.76		15.95	17.55	20.03		15.80	17.08		17.73	15.81		17.44	18.09
SeII	22.05	21.13	21.09		16.82	18.86	20.44		21.23	17.75		25.58	19.72		19.30	22.07
Average	17.93	18.58	19.19		16.21	19.57	19.45		16.91	17.60		19.36	17.06		18.62	19.39
*r*	0.764	0.943	0.141		0.845	−0.691	0.942		0.913	−0.287		0.916	0.801		0.140	0.828
LSD_0.05_	A—1.20, B—1.20, C—n.s., A × B—2.76, A × C—n.s., B × C—n.s., A × B × C—n.s.															
Common wheat																
1st selenium application date (C)																
Se0	17.07	22.28	21.21		16.93	19.99	17.92		21.05	21.66		22.34	17.71		21.07	20.22
SeI	15.96	19.86	18.99		16.77	19.59	18.43		15.47	18.57		18.20	16.04		19.32	18.59
SeII	13.32	16.05	18.50		14.65	12.99	16.97		17.44	14.96		18.50	15.00		14.49	17.89
Average	15.29	19.30	19.43		15.72	17.02	17.70		17.61	17.78		19.59	16.16		18.05	18.91
*r*	−0.973	−0.992	−0.938		−0.896	−0.890	−0.641		−0.638	−0.999		−0.832	−0.991		−0.965	−0.974
2nd selenium application date (C)																
Se0	17.07	22.28	21.21		16.93	19.99	17.92		21.05	20.66		22.34	17.71		21.07	20.22
SeI	16.03	18.02	17.36		15.35	16.07	18.51		14.81	15.58		16.93	15.35		16.51	17.63
SeII	17.08	16.73	16.62		17.71	17.43	18.83		16.77	15.19		18.75	17.13		16.46	18.03
Average	16.71	18.78	18.19		16.17	17.79	18.36		17.09	16.83		19.16	16.65		17.79	18.50
*r*	0.008	−0.955	−0.931		0.324	−0.643	0.986		−0.671	−0.896		−0.652	−0.236		−0.871	−0.785
LSD_0.05_	A—n.s., B—1.48, C—n.s., A × B—3.40, A × C—n.s., B × C—n.s., A × B × C—n.s.															
**(b)**																
**Selenium Dose (B)**	**Sulphur Dose (A)**										**Average**			***r***		
	**S0**			**SI**				**SII**								
Spelt wheat																
1st selenium application date (C)																
Se0	18.15 ^bcde^			18.73 ^de^				16.89 ^abcde^			17.66			−0.670		
SeI	16.02 ^abc^			15.48 ^b^				15.63 ^ab^			15.28			−0.700		
SeII	18.62 ^de^			17.18 ^cde^				16.97 ^abcd^			17.46			−0.919		
Average	17.36			17.00				16.34			16.68			−0.986		
*r*	0.170			−0.477				0.053			−0.076					
2nd selenium application date (C)																
Se0	18.15 ^bcde^			18.79 ^de^				16.60 ^abcde^			17.66			−0.688		
SeI	16.78 ^bcde^			17.84 ^bcde^				16.87 ^abcde^			17.09			0.077		
SeII	21.42 f			18.71 ^de^				21.52 a			20.26			0.031		
Average	18.57			18.41				17.96			18.18			−0.964		
*r*	0.686			−0.076				0.889			0.769					
Average																
Se0	18.15			18.76				16.75			17.66			−0.680		
SeI	16.40			16.66				16.25			16.19			−0.362		
SeII	20.02			17.95				19.25			18.86			−0.370		
Average	18.19			17.79				17.41			17.57			−1.000		
*r*	0.516			−0.385				0.779			0.448					
LSD_0.05_	A—1.20, B—1.20, C—n.s., A × B—2.76, A × C—n.s., B × C—n.s., A × B × C—n.s.															
Common wheat																
1st selenium application date (C)																
Se0	20.19 ^bc^			18.28 ^ab^				21.69 ^c^			19.67			0.439		
SeI	18.27 ^ab^			18.26 ^ab^				17.41 ^ab^			17.99			−0.871		
SeII	15.96 ^a^			14.87 ^ab^				16.97 ^a^			15.79			0.481		
Average	18.01			16.81				18.33			17.71			0.200		
*r*	−0.999			−0.869				−0.905			−0.997					
2nd selenium application date (C)																
Se0	20.19 ^bc^			18.28 ^ab^				21.35 ^c^			19.67			0.374		
SeI	17.14 ^a^			16.64 ^a^				15.77 ^a^			16.49			−0.988		
SeII	16.81 ^a^			17.99 ^ab^				16.91 ^a^			17.20			0.076		
Average	17.89			17.44				17.69			17.65			−0.444		
*r*	−0.907			−0.166				−0.753			−0.740					
Average																
Se0	20.19			18.28				21.52			19.67			0.408		
SeI	17.71			17.45				16.59			17.24			−0.954		
SeII	16.39			16.43				16.94			16.50			0.900		
Average	18.09			17.39				18.35			17.80			0.257		
*r*	−0.985			−0.998				−0.832			−0.956					
LSD_0.05_	A—n.s., B—1.48, C—n.s., A × B—3.40, A × C—n.s., B × C—n.s., A × B × C—n.s.															

*r*—correlation coefficient; LSD for A—sulphur dose, B—selenium dose, C—selenium application date; A × B, A × C, B × C, A × B × C—interactions; significant differences at *p* ≤ 0.05; n.s.—differences not significant. Values marked with the same letters do not differ significantly (*p* ≤ 0.05).

**Table 10:** (a). N:S ratio in straw of spelt wheat and common wheat. (b). N:S ratio in straw of spelt wheat and common wheat, averages for years.

**(a)**																
**Selenium Dose (B)**	**Sulphur Dose (A)**												**Average**			
	**S0**				**SI**				**SII**							
	**I Year**	**II Year**	**III Year**		**I Year**	**II Year**		**III Year**	**I Year**	**II Year**		**III Year**	**I Year**		**II Year**	**III Year**
Spelt wheat																
1st selenium application date (C)																
Se0	22.04	11.62	16.12		28.53	11.82		12.86	18.35	9.77		11.27	22.29		11.09	13.23
SeI	17.50	14.74	21.23		12.88	12.37		17.21	19.50	9.98		15.14	15.95		12.11	17.32
SeII	17.45	11.02	15.65		20.41	15.14		28.47	56.00	10.10		8.65	24.18		11.75	14.23
Average	19.00	12.31	17.43		18.06	12.85		18.04	23.16	9.88		11.37	20.11		11.47	14.77
*r*	−0.871	−0.150	−0.076		−0.519	0.933		0.969	0.879	0.988		−0.401	0.219		0.638	0.235
2nd selenium application date (C)																
Se0	22.04	12.06	16.12		28.53	11.82		12.86	18.35	9.77		11.27	22.29		11.09	13.23
SeI	27.14	6.62	19.62		20.59	14.13		18.68	29.47	11.00		13.11	25.43		10.22	16.97
SeII	19.30	13.91	16.87		14.53	18.00		19.68	18.32	9.94		16.26	16.82		13.25	17.53
Average	22.15	10.36	17.45		18.97	14.30		16.68	21.04	10.17		13.08	20.89		11.44	15.38
*r*	−0.344	0.244	0.203		−0.997	0.990		0.926	−0.002	0.128		0.989	−0.628		0.692	0.920
LSD_0.05_	A—n.s., B—n.s., C—n.s., A × B—n.s., A × C—n.s., B × C—n.s., A × B × C—n.s.															
Common wheat																
1st selenium application date (C)																
Se0	35.92	19.04	23.33		20.28	16.59		17.32	14.70	11.45		16.00	20.52		17.85	18.04
SeI	44.54	22.85	36.64		31.36	17.87		30.61	36.75	21.33		29.56	38.21		20.90	31.29
SeII	28.86	16.88	26.39		27.05	31.73		36.24	9.17	9.33		10.90	20.43		15.89	20.79
Average	40.00	19.33	28.50		30.76	20.23		27.55	14.94	15.24		16.16	24.55		18.00	22.70
*r*	−0.450	−0.357	0.219		0.606	0.902		0.974	−0.189	−0.166		−0.264	−0.004		−0.388	0.197
2nd selenium application date (C)																
Se0	35.92	19.04	23.33		20.28	16.59		17.32	14.70	11.45		16.00	20.52		17.85	18.04
SeI	43.08	16.68	35.56		29.06	19.09		28.00	19.60	18.28		19.60	32.76		18.16	27.71
SeII	20.59	9.10	12.45		25.93	15.91		16.45	40.47	25.20		26.94	26.46		13.74	16.68
Average	36.07	13.05	21.00		24.16	17.00		19.96	21.54	20.33		20.28	25.82		16.37	19.88
*r*	−0.667	−0.957	−0.471		0.635	−0.203		−0.068	0.942	1.000		0.981	0.485		−0.833	−0.113
LSD_0.05_	A—n.s., B—2.97, C—n.s., A × B—n.s., A × C—n.s., B × C—n.s., A × B × C—n.s.															
**(b)**																
**Selenium Dose (B)**	**Sulphur Dose (A)**										**Average**			***r***		
	**S0**			**SI**			**SII**									
Spelt wheat																
1st selenium application date (C)																
Se0	16.59 ^a^			17.74 ^a^			13.13 ^a^				14.03			−0.721		
SeI	17.82 ^a^			14.15 ^a^			14.87 ^a^				14.67			−0.758		
SeII	14.71 ^a^			21.34 ^a^			24.92 ^a^				15.63			0.985		
Average	16.25			16.32			14.80				14.59			−0.845		
*r*	−0.600			0.501			0.926				0.993					
2nd selenium application date (C)																
Se0	16.74 ^a^			17.74 ^a^			13.13 ^a^				14.10			−0.744		
SeI	17.79 ^a^			17.80 ^a^			17.86 ^a^				15.50			0.924		
SeII	16.69 ^a^			17.40 ^a^			14.84 ^a^				15.91			−0.700		
Average	16.66			16.65			14.76				15.26			−0.868		
*r*	−0.040			−0.788			0.357				0.954					
Average																
Se0	16.67			17.74			13.13				14.07			−0.733		
SeI	17.81			15.98			16.37				15.09			−0.747		
SeII	15.70			19.37			19.88				15.77			0.917		
Average	16.72			17.70			16.46				14.97			−0.203		
*r*	−0.458			0.480			1.000				0.994					
LSD_0.05_	A—n.s., B—n.s., C—n.s., A × B—n.s., A × C—n.s., B × C—n.s., A × B × C—n.s.															
Common wheat																
1st selenium application date (C)																
Se0	26.10 ^a^			18.06 ^a^			14.05 ^a^				19.28			−0.982		
SeI	34.68 ^a^			26.61 ^a^			29.21 ^a^				29.53			−0.664		
SeII	24.05 ^a^			31.67 ^a^			9.80 ^a^				18.86			−0.642		
Average	29.28			26.18			15.45				21.00			−0.953		
*r*	−0.182			0.989			−0.208				−0.035					
2nd selenium application date (C)																
Se0	26.10 ^a^			18.06 ^a^			14.05 ^a^				19.28			−0.982		
SeI	31.77 ^a^			25.38 ^a^			19.16 ^a^				25.29			−1.000		
SeII	14.05 ^a^			19.43 ^a^			30.87 ^a^				18.16			0.979		
Average	23.37			20.37			20.72				20.19			−0.808		
*r*	−0.666			0.176			0.975				−0.146					
Average																
Se0	26.10			18.06			14.05				19.28			−0.982		
SeI	33.23			26.00			24.19				27.41			−0.945		
SeII	19.05			25.55			20.34				18.51			0.187		
Average	26.13			23.20			19.52				21.73			−0.998		
*r*	−0.497			0.840			0.614				−0.078					
LSD_0.05_	A—n.s., B—2.97, C—n.s., A × B—n.s., A × C—n.s., B × C—n.s., A × B × C—n.s.															

*r*—correlation coefficient; LSD for A—sulphur dose, B—selenium dose, C—selenium application date; A × B, A × C, B × C, A × B × C—interactions; significant differences at *p* ≤ 0.05; n.s.—differences not significant. Values marked with the same letters do not differ significantly (*p* ≤ 0.05).

## Data Availability

Data are contained within the article.

## References

[1] Malagoli M., Schiavon M., Dall’Acqua S., Pilon-Smits E.A.H. (2015). Effects of Selenium Biofortification on Crop Nutritional Quality. Front. Plant Sci..

[2] Zhou X., Yang J., Kronzucker H.J., Shi W. (2020). Selenium Biofortification and Interaction with Other Elements in Plants: A Review. Front. Plant Sci..

[3] Guo Q., Ye J., Zeng J., Chen L., Korpelainen H., Li C. (2023). Selenium Species Transforming along Soil–Plant Continuum and Their Beneficial Roles for Horticultural Crops. Hortic. Res..

[4] Liao Q., Xing Y., Li A.-M., Liang P.-X., Jiang Z.-P., Liu Y.-X., Huang D.-L. (2024). Enhancing Selenium Biofortification: Strategies for Improving Soil-to-Plant Transfer. Chem. Biol. Technol. Agric..

[5] Wang M., Zhou F., Cheng N., Chen P., Ma Y., Zhai H., Qi M., Liu N., Liu Y., Meng L. (2022). Soil and Foliar Selenium Application: Impact on Accumulation, Speciation, and Bioaccessibility of Selenium in Wheat (*Triticum aestivum* L.). Front. Plant Sci..

[6] Pei W., Dai M., Shi S., Zhang Y., Wu D., Qiao C., Sun Y., Wang J. (2025). Effects of Foliar Selenium Spraying on the Growth and Selenium Content and Morphology of Rice. Front. Plant Sci..

[7] Deng G., Fan Z., Wang Z., Peng M. (2025). Dynamic Role of Selenium in Soil–Plant–Microbe Systems: Mechanisms, Biofortification, and Environmental Remediation. Plant Soil.

[8] Jiang Z., Wang Z., Zhao Y., Peng M. (2024). Unveiling the Vital Role of Soil Microorganisms in Selenium Cycling: A Review. Front. Microbiol..

[9] Tolu J., Bouchet S., Helfenstein J., Hausheer O., Chékifi S., Frossard E., Tamburini F., Chadwick O.A., Winkel L.H.E. (2022). Understanding Soil Selenium Accumulation and Bioavailability through Size-Resolved and Elemental Characterization of Soil Extracts. Nat. Commun..

[10] Sekhurwane M., Tóth B., Moloi M.J. (2025). Effectiveness of Soil, Foliar, and Seed Selenium Applications in Modulating Physio-Biochemical and Yield Responses to Drought Stress in Vegetable Soybean (*Glycine max* L. Merrill). Plants.

[11] Broadley M.R., Alcock J., Alford J., Cartwright P., Foot I., Fairweather-Tait S.J., Hart D.J., Hurst R., Knott P., McGrath S.P. (2010). Selenium Biofortification of High-Yielding Winter Wheat (*Triticum aestivum* L.) by Liquid or Granular Se Fertilization. Plant Soil.

[12] Hart D.J., Fairweather-Tait S.J., Broadley M.R., Dickinson S.J., Foot I., Knott P., McGrath S.P., Mowat H., Norman K., Scott P.R. (2011). Selenium Concentration and Speciation in Biofortified Flour and Bread: Retention of Selenium During Grain Biofortification, Processing and Production of Se-Enriched Food. Food Chem..

[13] Curtin D., Hanson R., Van der Weerden T.J. (2008). Effect of Selenium Fertiliser Formulation and Rate of Application on Selenium Concentrations in Irrigated and Dryland Wheat (*Triticum aestivum*). N. Z. J. Crop Hortic. Sci..

[14] Alfthan G., Eurola M., Ekholm P., Venäläinen E.-R., Root T., Korkalainen K., Hartikainen H., Salminen P., Hietaniemi V., Aspila P. (2015). Effects of Nationwide Addition of Selenium to Fertilizers on Foods, and Animal and Human Health in Finland: From Deficiency to Optimal Selenium Status of the Population. J. Trace Elem. Med. Biol..

[15] Eurola M., Alainen T., Berlin T., Ekholm P., Erlund I., Hietaniemi V., Mannio J., Mykkänen S., Pulkkinen M., Root T. Report of the Selenium Working Group 2022. Natural Resources Institute Finland. http://urn.fi/URN:ISBN:978-952-380-534-7.

[16] CFIA (2022). Safety Standards for Fertilizers and Supplements. Canadian Food Inspection Agency. https://inspection.canada.ca.

[17] Yara International (2011). Selenium Fortified Fertilizers in Finland. https://farmingfirst.org/2011/01/selenium-fortified-fertilizers-in-finland.

[18] Ramkissoon C., Degryse F., da Silva R.C., Baird R., Young S.D., Bailey E.H., McLaughlin M.J. (2019). Improving the Efficacy of Selenium Fertilizers for Wheat Biofortification. Sci. Rep..

[19] Ratnasekera D., Bandara D.M.A.D., Madushanka K.S.J., Gunasekera D., Hemantha H.H. (2025). Modes of Selenium Application in Agriculture: Efficacy and Issues. Selenium in Sustainable Agriculture: A Soil to Spoon Prospective.

[20] Hu C., Nie Z., Shi H., Peng H., Li G., Liu H., Li C., Liu H. (2023). Selenium Uptake, Translocation, Subcellular Distribution and Speciation in Winter Wheat in Response to Phosphorus Application Combined with Three Types of Selenium Fertilizer. BMC Plant Biol..

[21] Yeasmin M., Lamb D., Choppala G., Rahman M.M. (2022). Impact of Sulfur on Biofortification and Speciation of Selenium in Wheat Grain Grown in Selenium-Deficient Soils. J. Soil Sci. Plant Nutr..

[22] Coppa E., Celletti S., Sestili F., Mimmo T., Garcia Molina M.D., Cesco S., Astolfi S. (2023). Interaction between Sulfate and Selenate in Tetraploid Wheat (*Triticum turgidum* L.) Genotypes. Int. J. Mol. Sci..

[23] El Mehdawi A.F., Jiang Y., Guignardi Z.S., Esmat A., Pilon M., Pilon-Smits E.A.H., Schiavon M. (2018). Influence of Sulfate Supply on Selenium Uptake Dynamics and Expression of Sulfate/Selenate Transporters in Selenium Hyperaccumulator and Nonhyperaccumulator *Brassicaceae*. New Phytol..

[24] Schiavon M., Pilon M., Malagoli M., Pilon-Smits E.A.H. (2015). Exploring the Importance of Sulfate Transporters and ATP Sulphurylases for Selenium Hyperaccumulation—A Comparison of *Stanleya pinnata* and *Brassica juncea* (*Brassicaceae*). Front. Plant Sci..

[25] de Souza Cardoso A.A., de Lima Gomes F.T., Antonio J.R.R., Guilherme L.R.G., Liu J., Li L., de Souza Silva M.L. (2022). Sulfate Availability and Soil Selenate Adsorption Alleviate Selenium Toxicity in Rice Plants. Environ. Exp. Bot..

[26] Jiang H., Lin W., Jiao H., Liu J., Chan L., Liu X., Wang R., Chen T. (2021). Uptake, Transport, and Metabolism of Selenium and Its Protective Effects against Toxic Metals in Plants: A Review. Metallomics.

[27] Gupta M., Gupta S. (2017). An Overview of Selenium Uptake, Metabolism, and Toxicity in Plants. Front. Plant Sci. Sec. Plant Membr. Traffic Transp..

[28] Pilon-Smits E.A.H., Quinn C.F., Hell R., Mendel R.-R. (2010). Selenium Metabolism in Plants. Cell Biology of Metals and Nutrients.

[29] Trippe R.C., Pilon-Smits E.A.H. (2021). Selenium Transport and Metabolism in Plants: Phytoremediation and Biofortification Implications. J. Hazard. Mater..

[30] White P.J., Bowen H.C., Parmaguru P., Fritz M., Spracklen W.P., Spiby R.E., Meacham M.C., Mead A., Harriman M., Trueman L.J. (2004). Interactions Between Selenium and Sulphur Nutrition in *Arabidopsis thaliana*. J. Exp. Bot..

[31] Barak P., Goldman I.L. (1997). Antagonistic Relationship Between Selenate and Sulfate Uptake in Onion (*Allium cepa*): Implications for the Production of Organosulfur and Organoselenium Compounds in Plants. J. Agric. Food Chem..

[32] Sors T.G., Ellis D.R., Salt D.E. (2005). Selenium Uptake, Translocation, Assimilation and Metabolic Fate in Plants. Photosynth. Res..

[33] Saeed K., Nisa F.K., Abdalla M.A., Mühling K.H. (2023). The Interplay of Sulfur and Selenium Enabling Variations in Micronutrient Accumulation in Red Spinach. Int. J. Mol. Sci..

[34] Brodowska M.S., Kurzyna-Szklarek M., Wyszkowski M. (2025). Sulphur and Selenium as Determinants of Yield and Biometric Parameters in Wheat. Agronomy.

[35] Lara T.S., Correia T.S., de Oliveira C., Lessa J.H.d.L., de Souza K.R.D., Corguinha A.P.B., da Silva E.C., Martins F.A.D., Lopes G., Guilherme L.R.G. (2024). Selenium Application Provides Nutritional and Metabolic Benefits to Wheat Plants. Agronomy.

[36] Zhang D., Liu J., Cheng T., Wang H., Zhou Y., Gong Z., Hu T. (2025). Strategic Selenium Application Methods and Timing Enhance Grain Yield, Minimize Cadmium Bioaccumulation, and Optimize Selenium Fortification in *Triticum aestivum* L. Agronomy.

[37] Brodowska M.S., Kurzyna-Szklarek M., Wyszkowski M. (2025). Selenium and Sulphur as Elements Modifying Plant Quality: Assessment of the Content of Organic and Mineral Nitrogen Forms in Wheat. Molecules.

[38] Zhou Y., Nie K., Geng L., Wang Y., Li L., Cheng H. (2025). Selenium’s Role in Plant Secondary Metabolism: Regulation and Mechanistic Insights. Agronomy.

[39] Jia S., Guan Q., Niu Y., Wang Y., Li L., Cheng H. (2025). Progress in Elucidating the Mechanism of Selenium in Mitigating Heavy Metal Stress in Crop Plants. Agriculture.

[40] Bin Daud M.F., Rempelos L., Çakmak I., Leifert C., Bilsborrow P. (2024). Increasing grain selenium concentration via genetic and agronomic innovations. Plant Soil.

[41] Brodowska M., Kurzyna-Szklarek M., Czeczko R. (2025). Evaluation of the effect of sulphur and selenium applications on copper and zinc content in spelt wheat (*Triticum spelta* L.) and common wheat (*Triticum aestivum* L.). J. Elementol..

[42] Sun H., Lian X., Yao R., Shang B., Yi S., Yu J., Zhang B., Wang X. (2025). Synthesis, Antibacterial Properties, and Physiological Responses of Nano-Selenium in Barley (*Hordeum vulgare* L.) Seedlings Under Cadmium Stress. Agronomy.

[43] Burmistrov D.E., Shumeyko S.A., Semenova N.A., Dorokhov A.S., Gudkov S.V. (2025). Selenium Nanoparticles (Se NPs) as Agents for Agriculture Crops with Multiple Activity: A Review. Agronomy.

[44] González-Morales S., Pérez-Labrada F., García-Enciso E.L., Leija-Martínez P., Medrano-Macías J., Dávila-Rangel I.E., Juárez-Maldonado A., Rivas-Martínez E.N., Benavides-Mendoza A. (2017). Selenium and Sulfur to Produce *Allium* Functional Crops. Molecules.

[45] Kopriva S., Rahimzadeh Karvansara P., Takahashi H. (2024). Adaptive Modifications in Plant Sulfur Metabolism over Evolutionary Time. J. Exp. Bot..

[46] Suran P., Balík J., Kulhánek M., Sedlář O., Černý J. (2023). Influence of Long-Term Organic Fertilization on Changes in the Content of Various Forms of Sulfur in the Soil under Maize Monoculture. Agronomy.

[47] Magnucka E.G., Kulczycki G., Oksińska M.P., Kucińska J., Pawęska K., Milo Ł., Pietr S.J. (2023). The Effect of Various Forms of Sulfur on Soil Organic Matter Fractions and Microorganisms in a Pot Experiment with Perennial Ryegrass (*Lolium perenne* L.). Plants.

[48] Radawiec A., Szulc W., Rutkowska B. (2021). Selenium Biofortification of Wheat as a Strategy to Improve Human Nutrition. Agriculture.

[49] Wang M., Li B., Li S., Song Z., Kong F., Zhang X. (2021). Selenium in Wheat from Farming to Food. ACS J. Agric. Sci. Technol..

[50] Ducsay L., Zapletalová A., Slepčan M., Vicianová M., Hozlár P., Bušo R. (2021). Selenium Effect on Wheat Grain Yield and Quality Applied in Different Growth Stages. Plant Soil Environ..

[51] Manojlović M.S., Lončarić Z., Cabilovski R.R., Popović B., Karalić K., Ivezić V., Ademi A., Singh B.R. (2019). Biofortification of Wheat Cultivars with Selenium. Acta Agric. Scand. Sect. B Soil Plant Sci..

[52] Ngigi P.B., Lachat C., Masinde P.W., Du Laing G. (2019). Agronomic Biofortification of Maize and Beans in Kenya through Selenium Fertilization. Environ. Geochem. Health.

[53] Wang Q., Yu Y., Li J., Wan Y., Huang Q., Guo Y., Li H. (2017). Effects of Different Forms of Selenium Fertilizers on Se Accumulation, Distribution, and Residual Effect in Winter Wheat-Summer Maize Rotation System. J. Agri. Food Chem..

[54] Ducsay L., Ložek O., Marček M., Varényiová M., Hozlár P., Lošak T. (2016). Possibility of Selenium Biofortification of Winter Wheat Grain. Plant Soil Environ..

[55] Wang M., Ali F., Wang M., Dinh Q.T., Zhou F., Bañuelos G.S., Liang D. (2020). Understanding Boosting Selenium Accumulation in Wheat (*Triticum aestivum* L.) Following Foliar Selenium Application at Different Stages, Forms, and Doses. Environ. Sci. Pollut. Res..

[56] Klikocka H., Kobiałka A., Szostak B., Barczak B. (2017). Effect of Sulphur and Nitrogen Fertilization on the Selenium Content and Uptake by Grain of Spring Wheat. J. Elementol..

[57] Xia Q., Yang Z.P., Xue N.W., Dai X.J., Zhang X., Gao Z.Q. (2019). Effect of Foliar Application of Selenium on Nutrient Concentration and Yield of Colored-Grain Wheat in China. Appl. Ecol. Environ. Res..

[58] Gestal Reis H.P., de Queiroz Barcelos J.P., Furlani Junior E., Santos E.F., Silva V.M., Moraes M.F., Putti F.F., dos Reis A.R. (2018). Agronomic Biofortification of Upland Rice with Selenium and Nitrogen and its Relation to Grain Quality. J. Cereal Sci..

[59] Gao F., Wang L., Zhao R., Wang Y., Ma Y., Yang R., Zhang Q., Wang C. (2024). Rational Combination of Selenium Application Rate and Planting Density to Improve Selenium Uptake, Agronomic Traits, and Yield of Dryland Maize. Plants.

[60] Bañuelos G.S., Freeman J., Arroyo I. (2020). Accumulation and Speciation of Selenium in Biofortified Vegetables Grown Under High Boron and Saline Field Conditions. Food Chem. X.

[61] Galić L., Vinković T., Ravnjak B., Lončarić Z. (2021). Agronomic Biofortification of Significant Cereal Crops with Selenium—A Review. Agronomy.

[62] Borthakur U., Pradhan A.K., Sarma N., Jyoti S.Y., Kalita S.S., Kundu B., Poudel J., Tanti B. (2025). Role of Selenium in Biofortification of Cereals. Selenium in Sustainable Agriculture: A Soil to Spoon Prospective.

[63] NIH Selenium—Fact Sheet for Consumers. National Institutes of Health, Office of Dietary Supplements, Bethesda, Maryland, USA. https://ods.od.nih.gov/factsheets/Selenium/healthProfessional/.

[64] Estarriaga-Navarro S., Goicoechea N., Plano D., Sanmartín C. (2025). Selenium Biofortification: Integrating One Health and Sustainability. J. Sci. Food Agric..

[65] Roa G.A., Quintana-Obregón E.A., González-Renteria M., Ruiz Diaz D.A. (2024). Increasing Wheat Protein and Yield through Sulfur Fertilization and its Relationship with Nitrogen. Nitrogen.

[66] Liu Z., Liu D., Fu X., Du X., Zhang Y., Zhen W., Li S., Yang H., He S., Li R. (2022). Integrated Transcriptomic and Metabolomic Analyses Revealed the Regulatory Mechanism of Sulfur Application in Grain Yield and Protein Content in Wheat (*Triticum aestivum* L.). Front. Plant Sci..

[67] Azad M.A.K., Ahmed T., Eaton T.E.-J., Hossain M.M., Haque M.K., Soren E.B. (2021). Yield of Wheat (*Triticum aestivum*) and Nutrient Uptake in Grain and Straw as Influenced by Some Macro (S & Mg) and Micro (B & Zn) Nutrients. Nat. Sci..

[68] Hrivna L., Kotková B., Burešová I. (2015). Effect of Sulphur Fertilization on Yield and Quality of Wheat Grain. Cereal Res. Commun..

[69] Stankowski S., Podolska G., Kaczmarek S., Jaroszewska A., Hury G., Sobolewska M. (2019). Influence of Sulphur Fertilization on Yielding and Chemical Composition of Grain of Spring Wheat (*Triticum aestivum* L.) Grown in Different Habitat Conditions. J. Elementol..

[70] Rodrigo S., Santamaría O., López-Bellido F.J., Poblaciones M.J. (2013). Agronomic Selenium Biofortification of Two-Rowed Barley under Mediterranean Conditions. Plant Soil Environ..

[71] Silva V.M., Wilson L., Young S.D., Broadley M.R., White P.J., dos Reis A.R. (2023). Interaction between Sulfur and Selenium in Agronomic Biofortification of Cowpea Plants under Field Conditions. Plant Soil.

[72] LaBarge G. *Sulfur and Nitrogen Management in Wheat*; Ohio Ag Net Text Alerts: Columbus, OH, USA, 2025. https://ocj.com/2025/02/sulfur-and-nitrogen-management-in-wheat/.

[73] Schulte E.E., Kelling K.A. (2014). Soil and Applied Sulfur. University of Wisconsin—Madison Exten. Bull. A2525.

[74] Pokhrel D., Neupane R., Aryal S., Panth S., Khanal B. (2023). Effect of Different Doses of Sulfur on Growth and Yield of Rapeseed (*Brassica campestris* var. Lumle Tori). AgroEnviron. Sustain..

[75] Gil-Díaz M., Alonso J., Mancho C., García-Gonzalo P., Lobo M.C. (2024). Sulfur Induces Arsenic Tolerance in Barley Plants. Agriculture.

[76] Qiu C.-W., Dawood M., Zhao J., Chen Z.-H., Wu F. (2025). Differential Physiological and Proteomic Responses of Barley Genotypes to Sulfur Availability. Plant Growth Regul..

[77] Lima Gomes F.T.d., Chales A.S., Borghi E.J.A., Ferreira A.C.M., Souza B.C.O.Q.d., Nascimento V.L., Silva M.L.S. (2025). Agronomic Biofortification with Selenium and Zinc in Tomato Plants (*Solanum lycopersicum* L.) and Their Effects on Nutrient Content and Crop Production. J. Soil Sci. Plant Nutr..

[78] Boldrin P.F., de Figueiredo M.A., Yang Y., Luo H., Giri S., Hart J.J., Faquin V., Guilherme L.R.G., Thannhauser T.W., Li L. (2016). Selenium Promotes Sulfur Accumulation and Plant Growth in Wheat (*Triticum aestivum*). Physiol. Plant..

[79] Fleuridor L., Fulford A., Lindsey L.E., Lentz E., Watters H., Dorrance A., Minyo R., Richer E., Chaganti V., Subburayalu S. (2023). Ohio Grain Crop Response to Sulfur Fertilization. Agron. J..

[80] Mustafa A., Athar F., Khan I., Chattha M.U., Nawaz M., Shah A.N., Mahmood A., Batool M., Aslam M.T., Jaremko M. (2022). Improving Crop Productivity and Nitrogen Use Efficiency Using Sulfur and Zinc-Coated Urea: A Review. Front. Plant Sci. Sec. Crop Prod. Physiol..

[81] Talukdar S., Dutta P., Dutta J., Hussain J. (2022). Nitrogen and Sulphur Interaction on Nutrient Use Efficiency in Field Crops: A Review. Pharma Innov. J..

[82] Agyin-Birikorang S., Boubakry C., Adu-Gyamfi R., Chambers R.A., Fuseni A.-R.A., Kadyampakeni D.M. (2023). Synergism of Sulfur Availability and Agronomic Nitrogen Use Efficiency. Agron. J..

[83] Sedlár O., Balík J., Kulhánek M., Cerny J., Suran P. (2019). Sulphur Nutrition Index in Relation to Nitrogen Uptake and Quality of Winter Wheat Grain. Chil. J. Agric. Res..

[84] Grzebisz W., Potarzycki J. (2025). A Realistic Approach to Calculating the Nitrogen Use Efficiency Index in Cereals with Winter Wheat (*Triticum aestivum* L.) as an Example. Agronomy.

[85] Unger C., Flaten D.N., Lukow O.M., Grant C. Effect of N:S Ratio on the Breadmaking Quality of Wheat: Preliminary Findings from 1999. Soils and Crops Workshop, University of Saskatchewan, Canada, 107–120. https://harvest.usask.ca/server/api/core/bitstreams/ba375695-c849-405c-bada-e105de85e4cf/content.

[86] Klikocka H., Cybulska M., Barczak B., Narolski B., Szostak B., Kobiałka A., Nowak A., Wójcik E. (2016). The Effect of Sulphur and Nitrogen Fertilization on Grain Yield and Technological Quality of Spring Wheat. Plant Soil Environ..

[87] Arrigoni A.C., Lázaro L., Rogers W.J., Arata A.F., Tranquilli G.E. (2025). Incidence of Nitrogen Fertilization and Nitrogen/Sulfur Complementary Fertilization on Protein Accumulation, Yield, and End-Use Quality of Bread Wheat Cultivars. Cereal Res. Commun..

[88] Ullah I., Muhammad D., Musarat M. (2025). Effect of Various Nitrogen and Sulfur Sources on Maize-Wheat Yield and N:S Uptakes Under Two Different Climatic Conditions. Agric. Res..

[89] Chien S.H., Singh U., Ruiz Diaz D.A. (2021). Evaluation of Biosolids-Enriched Ammonium Sulfate vs. Ammonium Sulfate in Soils and for Plant Growth. Agron. J..

[90] Pimentel J.R., Troyjack C., Dubal Í.T.P., Peter M., Jaques L.B.A., Koch F., Carvalho I.R., dos Santos Bilhalva N., Dellagostin S.M., Demari G.H. (2021). Nitrogen (N) and Sulphate (S) Fertilization in Wheat Crop: Effect on the Vigor of Seeds Produced. Aust. J. Crop Sci..

[91] Brodowska M.S., Muszyński P., Haliniarz M., Brodowski R., Kowalczyk-Juśko A., Sekutowski T., Kurzyna-Szklarek M. (2018). Agronomic Aspects of Switchgrass Cultivation and Use for Energy Purposes. Appl. Ecol. Environ. Res..

[92] Bogusz P., Rusek P., Brodowska M.S. (2021). Suspension Fertilizers: How to Reconcile Sustainable Fertilization and Environmental Protection. Agriculture.

[93] Brodowska M.S., Wyszkowski M., Kordala N. (2022). Use of Organic Materials to Limit the Potential Negative Effect of Nitrogen on Maize in Different Soils. Materials.

[94] IUSS Working Group WRB (2015). World Reference Base for Soil Resources 2014; International Soil Classification System for Naming Soils and Creating Legends for Soil Maps; Update 2015. World Soil Resources Reports No. 106.

[95] Bielecki K., Kulczycki G. Modification of Butters-Chenery Method for Determination of Total Sulfur in Plants and Soil. Wrocław University of Environmental and Life Sciences, 2016. https://www.researchgate.net/publication/289336014.

[96] Kurmanbayeva A., Brychkova G., Bekturova A., Khozin I., Standing D., Yarmolinsky D., Sagi M. (2017). Determination of Total Sulfur, Sulfate, Sulfite, Thiosulfate, and Sulfolipids in Plants. Methods in Molecular Biology.

[97] Sugiyama N. (2024). The Accurate Measurement of Selenium Using On-Line Isotope Dilution with Triple Quadrupole ICP-MS. Agil. Technol. Appl. Note.

[98] TIBCO Software Inc. (2017). Statistica (Data Analysis Software System).

[99] Gurvich V., Naumova M. (2021). Logical Contradictions in the One-Way ANOVA and Tukey–Kramer Multiple Comparisons Tests with More Than Two Groups of Observations. Symmetry.

[100] Midway S.R., Robertson M., Flinn S., Kaller M. (2020). Comparing Multiple Comparisons: Practical Guidance for Choosing the Best Multiple Comparisons Test. PeerJ.

